# Molecular tweezer–peptide conjugates disrupt the protein–protein interaction between survivin and histone H3 essential in mitosis

**DOI:** 10.3762/bjoc.22.41

**Published:** 2026-03-27

**Authors:** Catherine Gsell, Philipp Rebmann, Karina Opara, Christine Beuck, Peter Bayer, David Bier, Ingrid R Vetter, Thomas Schrader

**Affiliations:** 1 University of Duisburg-Essen, Faculty of Chemistry, Universitätsstr. 7, 45117 Essen, Germany; 2 Max-Planck-Institute of Molecular Physiology, Department of Mechanistic Cell Biology, Otto-Hahn-Straße 11, 44227 Dortmund, Germany

**Keywords:** click reaction, chromosomal passenger complex, protein–protein interaction, mitosis, X-ray crystallography

## Abstract

Peptide-modified supramolecular tweezers, a promising new class of chemical tools, were designed and employed to inhibit the interaction of the BIR domain of human survivin, a member of the chromosomal passenger complex (CPC), with the phosphorylated histone H3 N-terminal peptide. Fluorescence polarization measurements revealed a nanomolar affinity of the BIR domain for the peptide-tweezer, depending on the presence of lysine residue 121, as proven by the K121A mutant of survivin. Two crystal structures of C-terminally truncated human survivin with the peptide-tweezer molecules demonstrated that the peptide moiety binds the BIR domain as expected from the well-known published crystal structures of survivin with various peptides, but the tweezer itself, surprisingly, was bound to a putative Ca^2+^ ion and the side chain of Pro26, corresponding to a previously unknown binding mode. Guided by the accessibility of survivin’s lysine residues in the CPC, a number of new promising peptide tweezers was synthesized, able to connect both binding sites on the protein.

## Introduction

**Figure 1:**
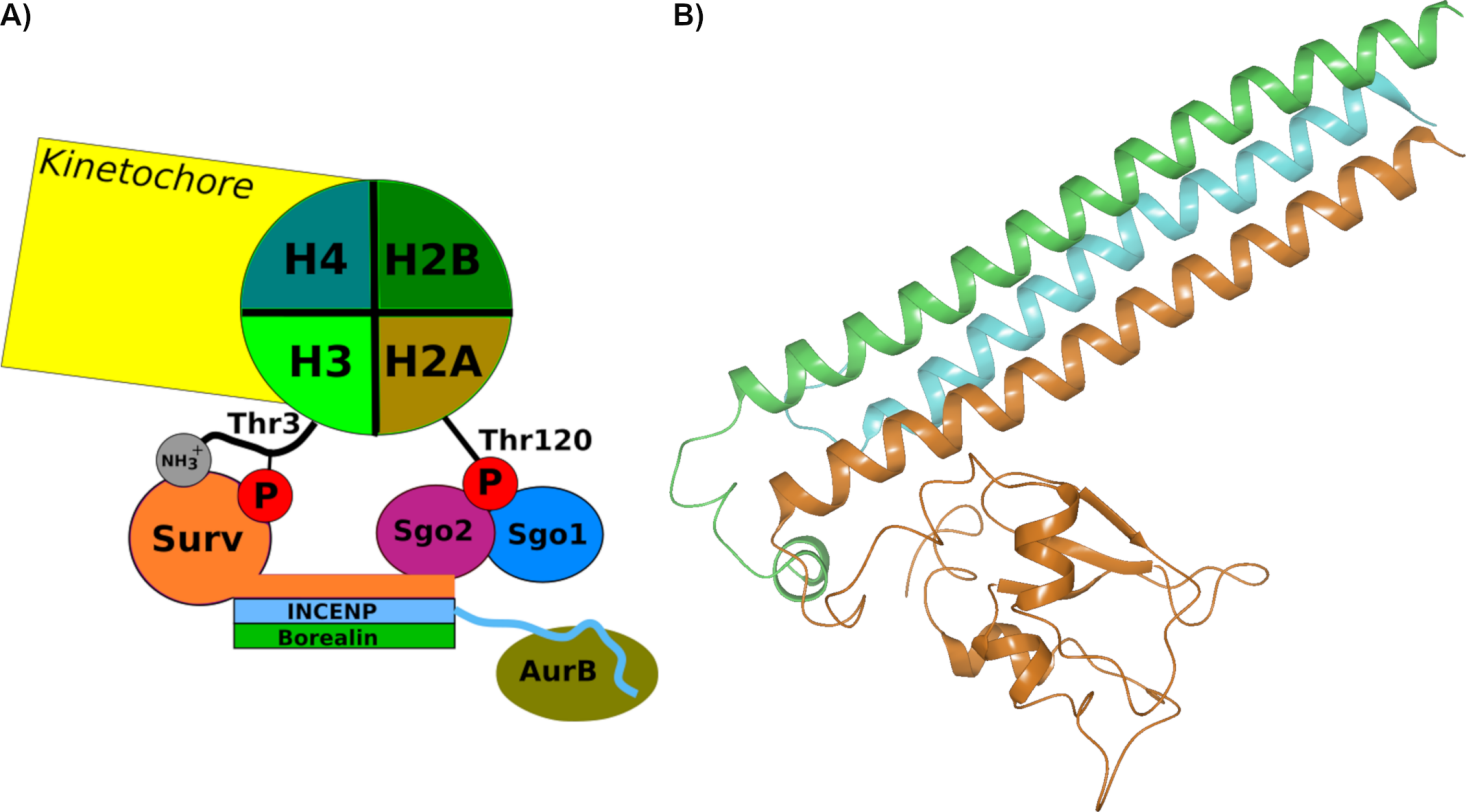
Mechanism of CPC recruitment to centromeres and kinetochores. A) Initially phosphomarks are placed on Thr3 of histone H3 and on Thr120 of histone H2A. In histone H3, P-Thr3 together with the free NH_3_^+^ of the first alanine residue contribute directly to the binding of survivin. P-T120 recruits shugoshin 1 and shugoshin 2, which in turn interact with borealin and with survivin itself, probably involving a region that is distinct from the one that interacts with P-T3-H3. B) Crystal structure of the CPC (PDB ID: 2QFA, https://doi.org/10.2210/pdb2QFA/pdb) [5] with the typical α-helix bundle from survivin (orange), borealin_10–109_ (green) und INCENP_1–58_ (blue).

The fundamental process of mitosis is controlled by a very large protein complex called the kinetochore, formed by self-assembly from hundreds of single protein components [1]. For the intricate regulation of the various phases of cell division, this kinetochore interacts with a smaller ensemble, the chromosomal passenger complex (CPC) that constitutes an abundant component of the inner centromere [2–3]. The CPC itself is formed inside the cell nucleus and consists of the four proteins survivin, borealin, INCENP (inner centromere protein), and the kinase Aurora B. During mitosis, Aurora B phosphorylates important components of the kinetochore and thus exerts control over key events of the whole process. The other three proteins localize the CPC during the different mitotic phases [4]. Survivin, borealin and INCENP are bound tightly to each other by aligning their extended α-helices (Figure 1) [5].

Intriguingly, CPC recruitment hinges on a few very distinct protein contacts, involving borealin and the BIR domain of survivin. A very dominant protein–protein interaction (PPI) is the embedding of the N-terminus of histone H3 on the BIR domain of survivin, which leads to attachment of the CPC to the inner centromere [6]. This PPI is facilitated by N-terminal H3 phosphorylation on Thr3, leading to recognition of the phosphorylated N-terminus by survivin and subsequent attachment of the CPC [7]. Jeyaprakash et al. demonstrated that survivin contributes most of the binding energy to this critical complex with histone H3 [8].

**Figure 2:**
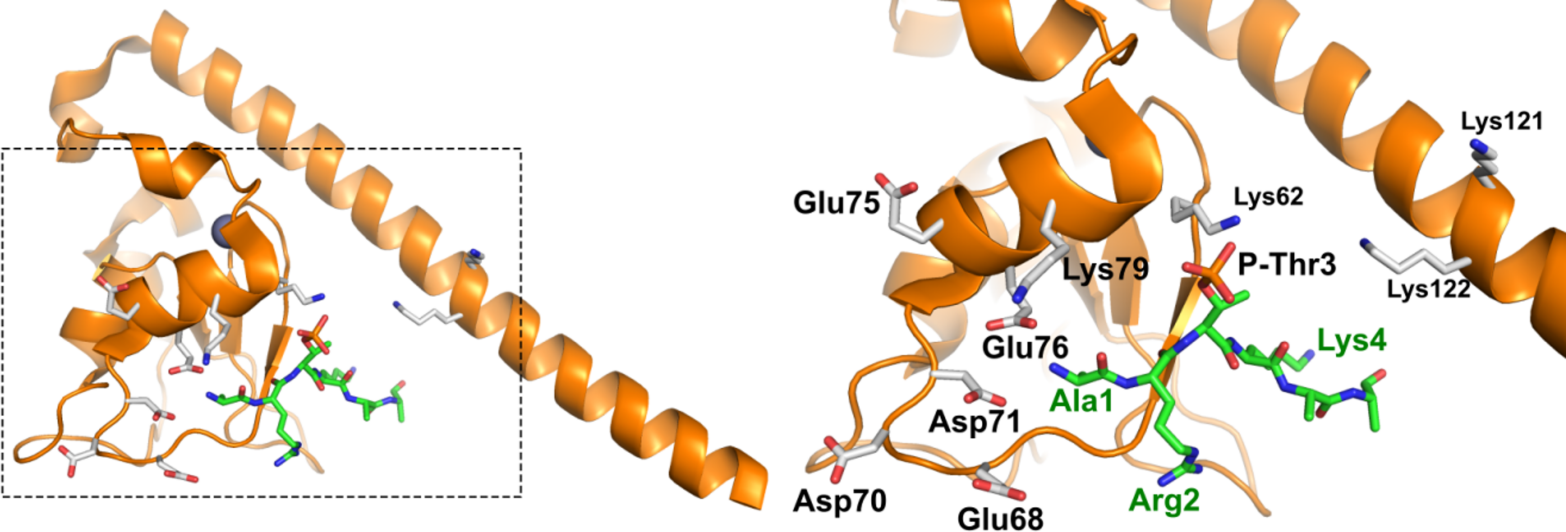
Ribbon model of crystal structure PDB ID 4A0J (https://doi.org/10.2210/pdb4A0J/pdb) [8] of survivin (orange) bound to the N-terminal region of histone H3 (green). The boxed region is enlarged in the right panel. Several of the residues lining the interface between survivin and histone H3 are shown.

In 2011, a crystal structure was solved depicting structural details of the H3 N-terminus (1–21) bound by survivin’s BIR domain (Figure 2). Only the first six residues (ARpTKQT) show electron density with well-defined localization on the survivin surface, all other amino acids are disordered [8]. This arrangement is stabilized by electrostatic interactions between histone H3’s phosphothreonine (pT) and basic residues on the core of the BIR domain as well as various basic residues of survivin’s α-helix. Likewise, histone’s cationic groups are distributed on a negatively charged survivin patch, while hydrophobic contacts are established towards both threonine and alanine methyl groups. This favorable natural arrangement was characterized by titration at ≈1 micromolar affinity [8].

A specific inhibitor of the survivin–histone H3 tail interaction would be a very valuable chemical tool because it could allow to deliberately shut down the recruitment of the CPC to the nucleosomes and to study its effect on CPC function.

## Results and Discussion

In 2005, our group discovered molecular tweezers with attached phosph(on)ate anions as powerful new hosts for the amino acids lysine and arginine. In the recent past, we demonstrated that the affinity and selectivity of our lysine-selective molecular tweezers could be improved by attaching natural peptide recognition elements. This could be documented in various projects with tailored peptide tweezer conjugates: For example, the self-complementary ELTLGEFL sequence of the nuclear export signal in survivin was coupled to tweezers which rendered them selective for this important interface (NMR evidence, 1:1 stoichiometry, K/A mutants) and prevented the survivin association with its export receptor CRM1 in pull-down assays with cell lysates [9]. Likewise, the ExoS interaction with its adapter protein 14-3-3 was effectively disrupted with a tweezer peptide conjugate that showed a 100-fold affinity increase and simultaneously occupied both an exposed lysine and precisely the natural binding site in the wide 14-3-3-cleft [10]. These examples for designed protein–protein interaction (PPI) inhibitors demonstrated that short but highly flexible linkers are imperative to increase selectivity and minimize entropical penalty. The design was strongly supported by computational modeling (MD and QM/MM simulations) as well as structural biology (NMR/X-ray).

Following our powerful concept of reinforcing natural peptide–protein interactions by tweezer conjugation [9–10], we envisaged to attach molecular tweezers to the histone H3 terminus at a distance which would allow a strong inclusion of a nearby R/K residue on survivin into the tweezer cavity. In fact five sterically well-accessible basic amino acids are located nearby in the survivin α-helix; their side chains point into the direction of the H3 binding site, ideally suited to accommodate the attached tweezers.

**Scheme 1:**
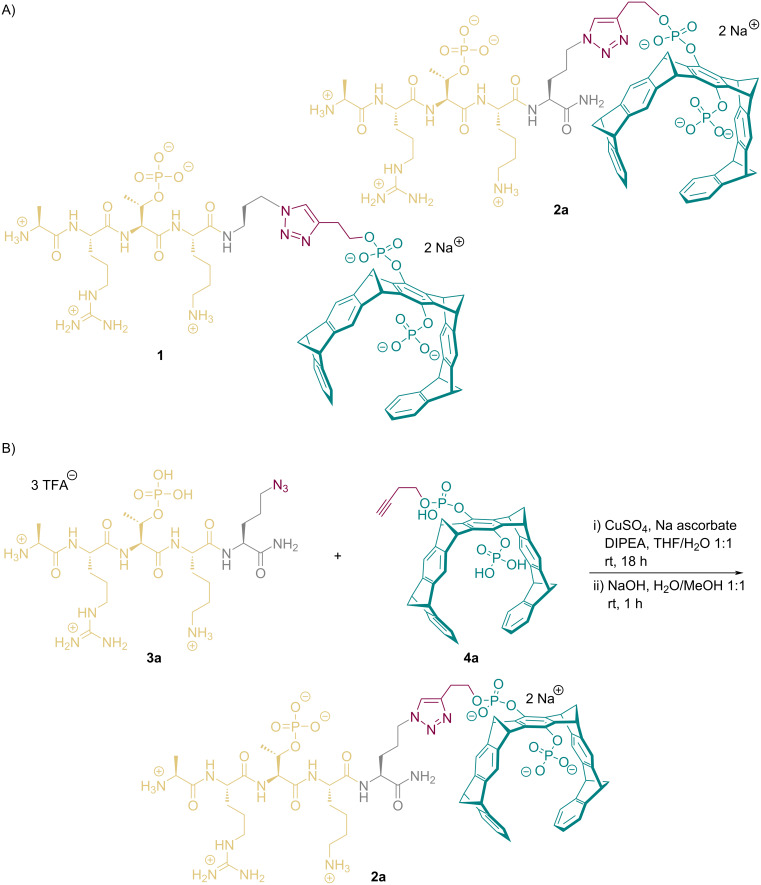
A) Peptide tweezer conjugates **1** and **2a** with triazoles linking the tweezer and H3 peptide at its C-terminus. Note that both azidopropylamine as well as azidoornithine establish almost the same distance between peptide and tweezer. In addition, they place the tweezer into the vicinity of the phosphorylated threonine, complementary to the cationic surface patch on survivin. B) Click coupling (i) between the H3-T3ph peptide with C-terminal 5-azidoornithine **3** and butynyl tweezer **4a** with subsequent deprotonation (ii) to the sodium salt of peptide tweezer **2a**. Binding peptide yellow, ornithine grey, butynyl group red, tweezer green.

Importantly, the H3 N-terminus had to be fully conserved *(*vide infra*)*, so that covalent attachment of the tweezer moiety had to occur at the peptide C-terminus. A first prototype **1** could be realized with a C3-spacer and click coupling between azidopropylamide on Lys-4 at the peptide's C-terminus and a monobutynyl tweezer (Scheme 1) [11]. Simultaneous placement of the peptide on its binding site and modelling of the tweezer moiety on Lys-121 produced favorable complex structures, which remained stable over 100 ns in MD simulations.

**Figure 3:**
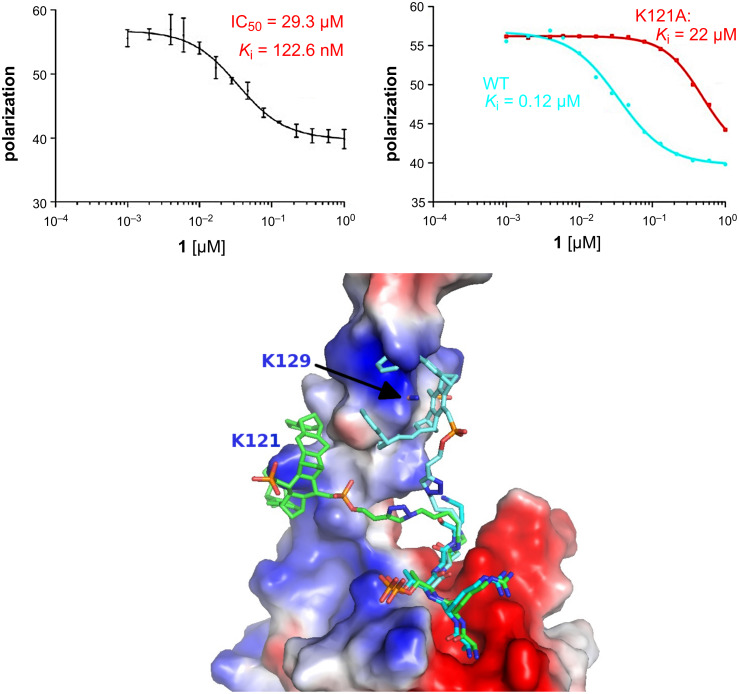
Survivin complexes with peptide tweezers: Fluorescence polarization measurements of wild type and mutated survivin (top) and modeling of the peptide tweezer **1** on either K-121 or K-129 (bottom). The positive residues of the H3 peptide are bound to the negatively charged area (red) whereas the phosphorylated threonine contacts a positively charged area, as known from the existing crystal structures. The tweezer moiety can associate with lysines in the C-terminal helix of survivin (positively charged blue area).

Competitive fluorescence polarization measurements between the tweezer H3 conjugate and a FITC-labeled H3 peptide pointed to very strong binding in the nanomolar range (Figure 3). Molecular modeling revealed that the tweezer moiety could easily reach Lys-121, but could bind to Lys-129 only in an energetically unfavorable, extended conformation. Indeed, mutation of Lys-121 to alanine in the survivin protein resulted in a drastic loss of binding affinity (WT 120 nM to K121A 22 µM) for the new ligand (Figure 3). We conclude that the new tweezer conjugate specifically directs its tweezer moiety to Lys-121 and thus reinforces the H3–survivin peptide–protein interaction by roughly one order of magnitude (from 1 µM to 120 nM *K*_d_).

For further structural proof we attempted co-crystallization of survivin with our new tweezer H3 conjugate. Unfortunately, crystallization of full length survivin 1–142 was difficult to reproduce and often resulted in low-quality crystals. Hence, we designed the truncation constructs 1–122, 1–127 and 1–134 that shorten the flexible C-terminal helix of survivin. All new constructs showed a much better expression and crystallization behavior. Fluorescence polarization measurements with the FITC-labeled H3 peptide confirmed that constructs 1–122, 1–127 as well as survivin full length (1–142) and the mutants K121A and K122A have the same affinity of approximately 1 μM to the peptide, i.e. the mutations/deletions do not influence the peptide binding site. It can therefore be expected that also with these survivin mutants there is an approximately 8-fold gain in affinity by attaching the tweezer moiety to the peptide, provided that the tweezer could reach a suitable binding partner as well.

To allow a general commercial synthesis of the phosphorylated H3-peptide sequences for the tweezers, we used an azidoornithine extension at the C-terminus (peptide ordered from Genscript), which was subjected to click reaction with the butynyl tweezer (Scheme 1). The coupling protocol was identical to the published version [9–10]. Reaction progress was monitored by analytical HPLC and the product could be purified by preparative HPLC. After lyophilization, the product **2a** was obtained as fully protonated TFA salt (43%); deprotonation with equimolar aq. NaOH produced the sodium salt of **2a** quantitatively.

The analytical characterization of **2a** turned out to be difficult, because even in DMSO-*d*_6_, most NMR signals remained very broad, and in part strongly shifted upfield. This may be explained by the three cationic groups in the peptide sequence, which likely interact with the negatively charged tweezer moiety by self-inclusion; it is well documented that these fast inclusion processes often lead to signal broadening and massive upfield shifts of the included side chain protons [12–13]. HPLC–HRMS analysis finally produced a single chromatographic signal with clean molecular ion peaks for [M + 2H]^2+^ and [M + 3H]^3+^. The high purity of **2a** was further demonstrated by successful co-crystallization of this tweezer peptide conjugate with survivin (vide infra).

Again, molecular docking of peptide and tweezer moiety **2a** on survivin led to stable arrangements on survivin, suggesting simultaneous occupation of the H3 binding site and potential complexation at Lys-121 (Figures S10 and S11 in Supporting Information File 1). The triazole linker may act as a barrier for the rearrangement of survivin’s side chains and the interaction diagram reveals contacts to all nine accessible acidic amino acids on the protein surface [14].

**Figure 4:**
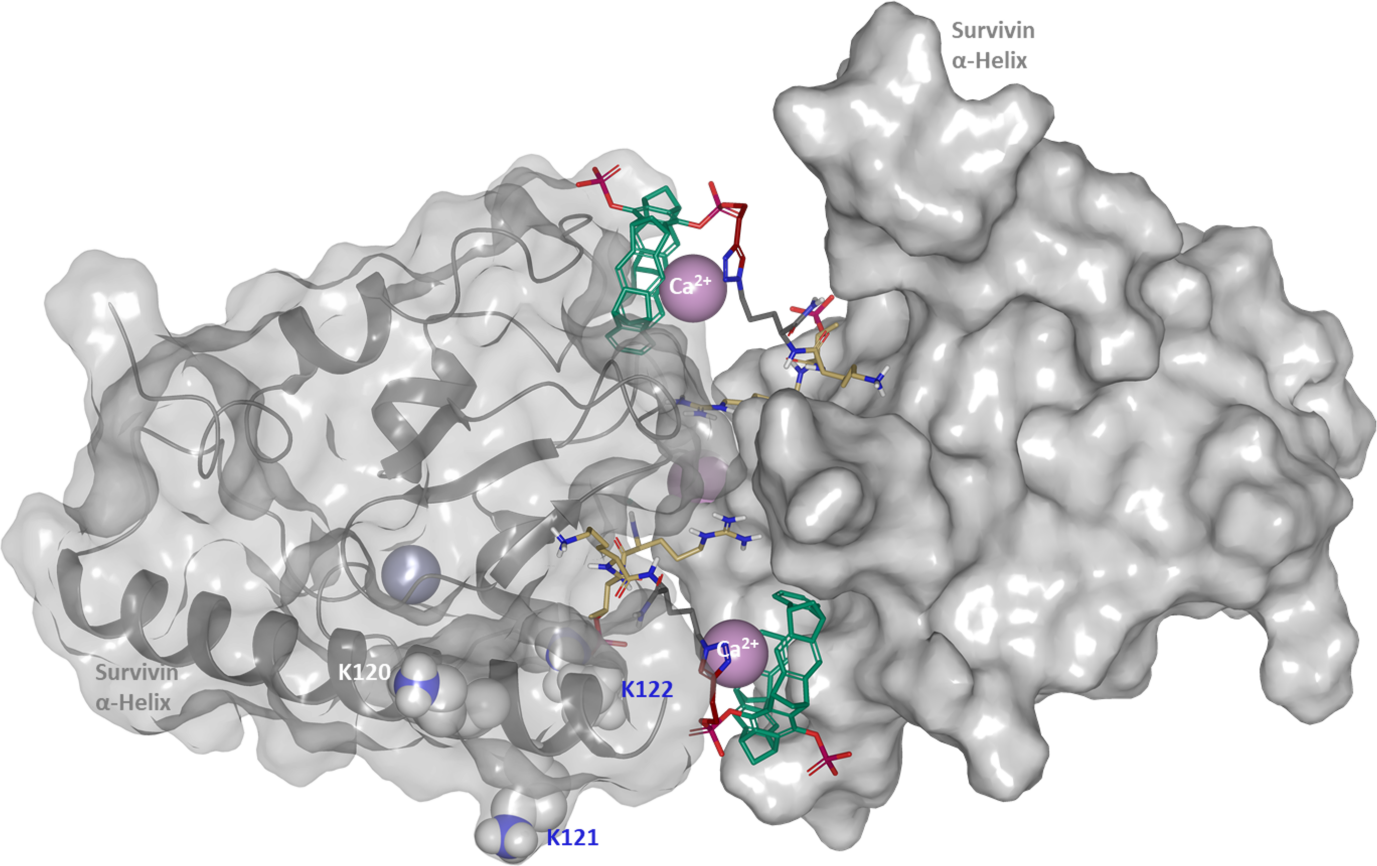
Crystal structure of the survivin dimer with complexed tweezer H3 conjugate **2a**. To distinguish both survivin molecules their surfaces (light grey) have been presented with a different degree of transparency. Lysines in the vicinity of the histone H3 binding as well as the Ca^2+^ ion site are labeled and shown as CPK models. The tweezer skeleton is shown in green, the H3 peptide in yellow.

The new tweezer H3 conjugate **2a** has a C4 spacer between the Lys-4 amide and the azide, very close to **1** with its C3 spacer. Crystallizations with this compound and the truncated survivin constructs 1–122 and 1–127 were finally successful and yielded high-resolution X-ray structures (Figure 4) [15]. The structures of both survivin truncation mutants look very similar, both have a survivin dimer in the asymmetric unit, and the H3 peptide binds in the canonical H3-peptide binding site with very well defined density. The tweezer moiety and the linker also have very well defined densities, but, contrary to our expectation, the tweezer does not bind to Lys-121, but instead packs against a proline of the second survivin monomer in the crystal (Figure 4 and Supporting Information File 1, Figure S12). Lys-121, on the other hand, is buried in a crystal contact. Thus, the crystal packing forces together with putatively competing ligands (see below) can apparently compete successfully with the tweezer that is most likely attached to Lys-121 in solution, as shown by the fluorescence polarization measurements where the K121A mutation has drastically reduced affinity.

In the new crystal structure, the apolar pyrrolidine of Pro-26 occupies half of the tweezer cavity, while from the opposite side, a density that most likely corresponds to a Ca^2+^ cation from the crystallization buffer, is visible next to the cavity (Figure S12 in Supporting Information File 1). From recent complexation experiments with lipids, it is known that even uncharged alkyl groups may occupy the tweezer cavity and gain binding energy through dispersive interactions and the hydrophobic effect [16]. Ca^2+^ may also interact with the high π-electron density inside the cavity, although in direct titrations, only small affinities in the millimolar range have been determined for this cation [17]. This observation not only indicates a competition of the (putative) calcium ion with amino acid side chains – that might also be in part be responsible for the lack of tweezer binding to Lys-121 – but also represents a new binding mode for the tweezer moiety. The (hydrated) Ca^2+^ ion is apparently too large to enter the cavity of the tweezer ring, in contrast to larger Cs^+^ ions that were surprisingly observed in the center of the aromatic ring structure, perhaps due to their weaker binding of the hydrate shell. However, this early crystal structure was obtained with the diacetoxytweezer in organic solution [18].

Interestingly, a similar unexpected inclusion of a methionine side chain was found in the crystal structure of the sulfonatocalix[4]arene (sclx4) with a trimeric RSL protein (Ralstonia solanacearum lectin, MK-RSL) that carried an engineered N-terminal Met-Lys extension [19], as well as in the protein P5CDH with a monophosphate-tweezer [20].

Close inspection of recent CPC crystal structures revealed partial burial of Lys-121 in the physiological surviving–borealin–INCENP complex (PDB ID 2QFA) [5], so that the tight alignment of three α-helices may also prevent efficient binding of our peptide tweezer to this seemingly favorable position. We therefore turned to peptide tweezers with longer linkers to address lysines which are buried neither in the crystals nor in the CPC complex. In a second approach, one of the tweezer phosphate groups was truncated to minimize steric hindrance during the tweezer approach towards survivin’s α-helix within the CPC.

Importantly, the peptide moiety itself cannot be modified substantially without losing affinity as described in the literature [8,21]. In most survivin–H3 peptide crystal structures, residues 1–4 have visible electron density, and only in few cases the following glutamine/threonine could be located as well (e.g., PDB ID 4A0J). The remainder of the peptides is usually disordered. Comparison of different peptides revealed that the N-terminal alanine is absolutely required for survivin binding, and no modification of the N-terminus is tolerated. Position 2 is probably uncritical, but most likely sensitive to the backbone conformation since it strongly influences the conformation of the neighboring alanine. Position 3 should either be a phosphothreonine or a glutamine, and position 4 is preferred as Arg or Lys. The peptide affinity drops 3- or 4-fold when increasing the pH from 6.8 to 8.2, thus limiting the crystallization conditions [22]. Other co-crystallized peptides are AKERC from the N-terminus of shugoshin1 (6.2 µM, PDB ID 4A0I) [8] and a Smac-DIABLO 15-mer-peptide with only 121 µM affinity, that still crystallized at sufficiently high concentrations in complex with survivin (PDB ID 3UIH https://doi.org/10.2210/pdb3UIH/pdb, [23]).

**Figure 5:**
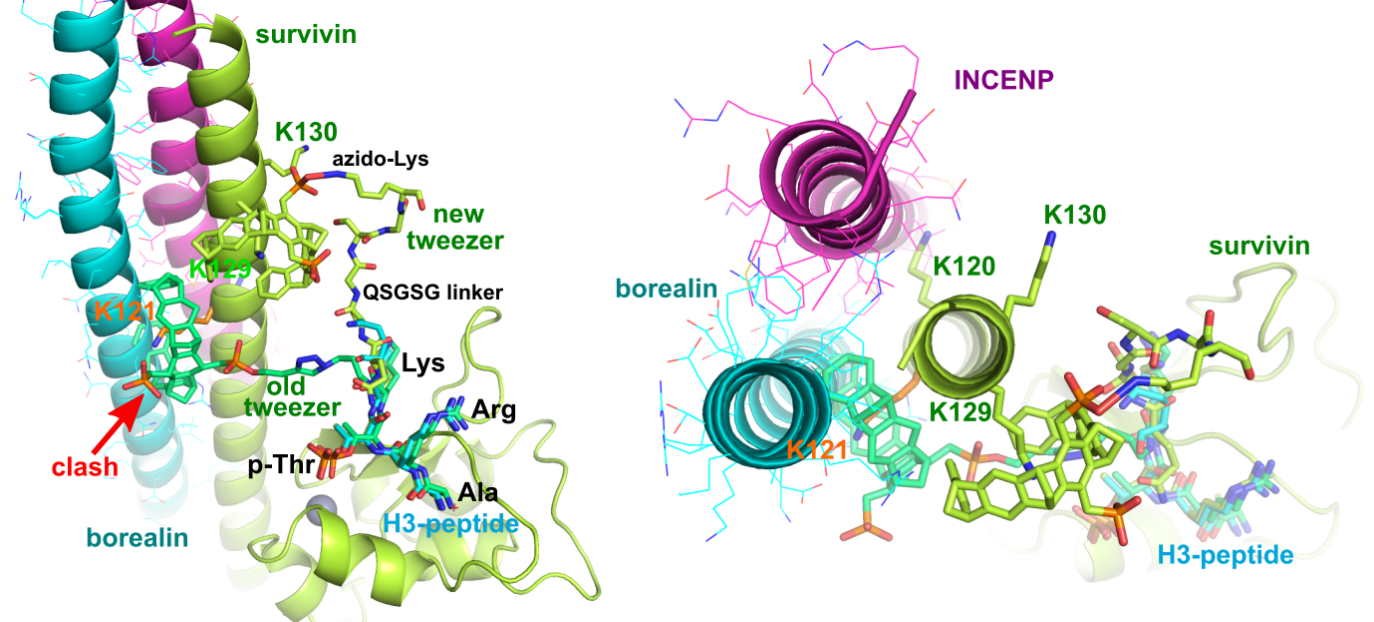
Survivin–borealin–INCENP complex structures (PDB ID 2QFA) [5] with tweezer model, side and top views. The original design ("old tweezer") could only reach Lys-121 which is not accessible in the CPC complex because it forms a tight trimer between survivin, borealin and INCENP. The newly designed compounds with longer linkers between the H3 peptide and the tweezer moiety could reach lysines 129 and 130 that are accessible even in the CPC complex.

A major problem of co-crystallizing tweezer compounds with proteins in general is the competition with the crystal packing, as illustrated by earlier crystal structures [20]. Since the "proof-of-concept" H3-peptide tweezer **1** targeted lysine 121 which is not only buried in the crystal, but also in the biological target, the CPC complex, two obvious design options remain: either introducing a shorter linker to address lysines closer to the peptide binding site and benefit from increased affinity due linker rigidity, or using a longer linker that could reach lysines 129 and 130 which are not buried in the known crystal forms, nor in the CPC complex (Figure 5). A 3D structure of the complex between the full CPC trimer and the hybrid H3-peptide–tweezer constructs would be very desirable to validate the binding mode in the physiological setting.

**New tweezer–H3 conjugates for well-accessible lysines in the CPC.** For efficient docking of a tweezer molecule onto a lysine residue on the protein surface, only one phosphate moiety is sufficient, which locks the included lysine cation into an ion pair [20]. In MD simulations between survivin and the truncated version of the tweezers conjugate **2b** (simply lacking the second phosphate) stable structures were produced which demonstrate the minimal steric demand of the remaining hydroquinone OH group, leaving much more room for tight contacts with the protein surface. In principle, a click reaction between the known unsymmetrical intermediate hydroxy-butynylphosphate tweezer and any azido histone peptide will lead to the desired truncated tweezer conjugate. This concept proved feasible and was performed with H3 peptides of different lengths to produce new peptide tweezer conjugates designed to target near and far lysines on survivin’s α-helix.

**Figure 6:**
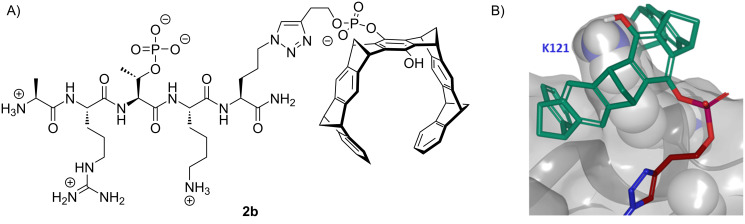
A) Lewis structure of the truncated tweezer peptide monophosphate **2b**. B) Close-up of the binding motif formed by **2b** (tweezer: green, butynyl ester: red) on the survivin surface encapsulating Lys-121 after MD simulation (Desmond, 100 ns, 300 K, 0.15 mM NaCl) using explicit water solvent.

As an example, Figure 6 depicts the Lewis structure of the tweezer conjugate **2b** from butynyl tweezer and the peptide. MD simulations used the new crystal structure of **2a** on survivin as starting point, and truncated one phosphate group to probe the importance of the respective phosphate ammonium ion pair. Interestingly, after 100 ns (Figure 6B) the tweezer cavity of **2b** still firmly rests on Lys-121, while the peptide remains in its binding groove. This result may reflect the comparable lysine affinities of mono- and diphosphate tweezers [24], and the compact complex structure of **2b** on K-121 leaves more room for stabilizing packing effects.

Lys-122 is also located in the vicinity of the H3-T3ph binding site; although it forms a protein contact with Asn-118, there is plenty of room around it in the crystal structure (Figure 4). However, to target this amino acid, the linker between tweezer and peptide must be considerably shortened; loss of Lys-4 would thus substantially lower the overall binding energy. Extended linkers on the other hand may allow complexation of well-accessible Lys-120, 129 or Lys-130. Just one more glycine placed between the H3 peptide and 5-azidoornithine furnishes tweezers **2c** and **2d** as mono- and diphosphate.

The small added glycine linker renders the peptide backbone more flexible, and allows the new tweezer to avoid clashes with crystal contacts. MD simulations also demonstrate that **2d** may indeed reach and rest upon Lys-120 (Figure S14 in Supporting Information File 1). Even more efficient should be the sterically compact monophosphate **2c**.

**Scheme 2:**
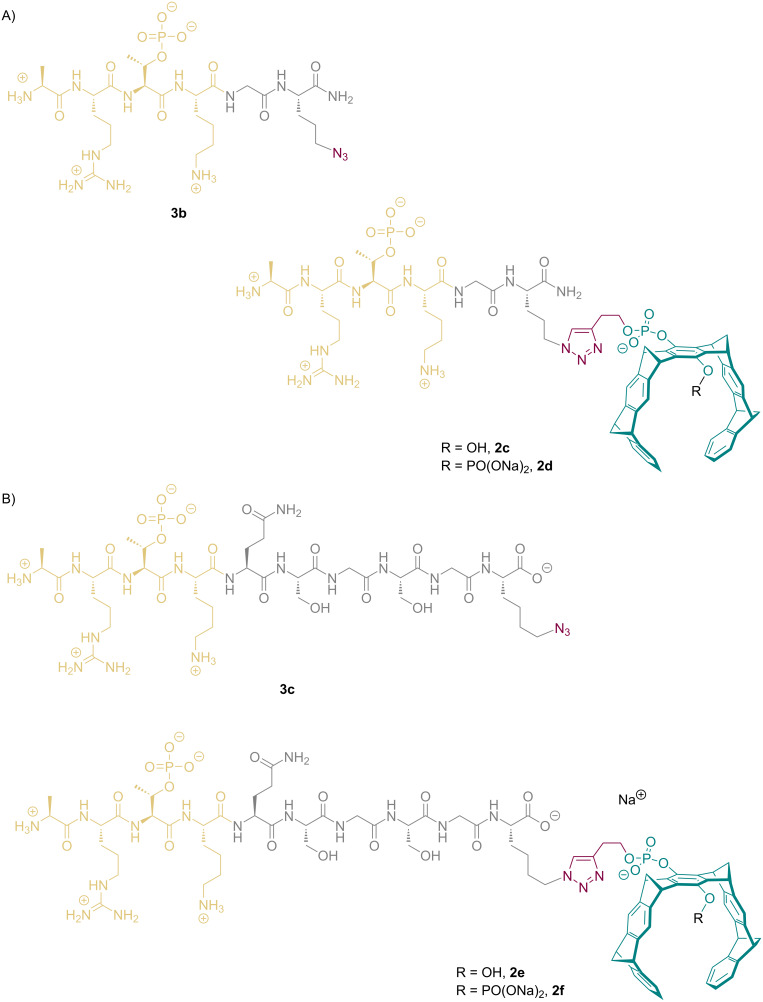
A) Lewis structures of the new slightly extended binding peptide **3b** and the respective click conjugates with butynyl tweezer, mono- and diphosphate **2c** and **2d**. B) Lewis structures of the new binding peptide **3c** and the resulting mono- and diphosphate tweezer conjugates **2e** and **2f**.

To reach Lys-139 and Lys-140, the linker must be elongated to a pentapeptide sequence. We decided to synthesize the peptide shown in Scheme 2B which features an Asn-Ser-Gly-Ser-Gly linker. The resulting tweezer peptide conjugates **2e** and **2f** are much more flexible and may reach lysines in larger distance, which did not show up in manual structure screens. Both tweezer conjugates **2e** and **2f** were synthesized, purified by preparative HPLC and analytically characterized. They are currently involved in crystallization experiments, competitive fluorescence titrations, and cell division assays.

## Conclusion

This report introduces a new supramolecular attempt to interfere with the critical interaction between kinetochore and CPC during mitosis in cells. The concept builds on the discovery that a specific protein–protein interaction (PPI) recruits the CPC to the kinetochore which hinges on its histone H3 peptide binding with its N-terminal sequence to the BIR domain of survivin. The biological affinity of this interaction (*K*_d_ ≈ 1 µM) is not sufficient as anchor point for an inhibitor; therefore a molecular tweezer selective for accessible lysine residues was covalently connected to the histone H3 peptide at various distances to add the released lysine inclusion energy (*K*_d_ ≈ 10 µM) and reach nanomolar affinity.

Azidopeptides were conjugated to butynyl tweezers by click chemistry and subjected to competitive fluorescence titrations with wildtype and mutant survivin as well as to co-crystallization experiments. These investigations demonstrated affinities of the peptide tweezers in the 100 nM range and proved that the peptide was placed inside its known binding site in two survivin crystal structures. However, the tweezer cavity did not include Lys-121 as planned but instead was engaged in a double interaction with Ca^2+^ and Pro-126, illustrating the variability of tweezer interactions. After extensive modeling using MD simulations with explicit solvent treatment, several new tweezer peptide conjugates were identified with highly promising complex structures on survivin that were stable for 100 ns.

Our new tweezer H3 conjugates **2a**–**f** target Lys-122, Lys-129, and Lys-130 which are freely accessible even in the CPC complex. Hence we expect them to bind with much enhanced specificity and affinity to survivin and prevent its binding to histone H3, thus creating an interesting tool to specifically interfere with this interaction in the cell. If these constructs are not sufficiently cell-permeable, they may be introduced via electroporation into mammalian cells, a protocol that is well established in the Musacchio lab [25]. Affinity determination, co-crystallization attempts, and bioassays are underway in our laboratories.

## Supporting Information

File 1 Experimental part.

## Data Availability

All data that supports the findings of this study is available in the published article and/or the supporting information of this article.
