# Supplementary material for: Molecular tweezer–peptide conjugates disrupt the protein–protein interaction between survivin and histone H3 essential in mitosis
Source: Beilstein J Org Chem. 2026 Mar 27;22:557–67. doi: 10.3762/bjoc.22.41 (PMC13040270; doi:10.3762/bjoc.22.41)
Supplement: File 1 — Experimental part. [file Beilstein_J_Org_Chem-22-557-s001.pdf]

BEILSTEIN JOURNAL OF ORGANIC CHEMISTRY

Supporting Information

for

Molecular tweezer–peptide conjugates disrupt the protein–protein interaction between survivin and histone H3 essential in mitosis

Catherine Gsell, Philipp Rebmann, Karina Opara, Christine Beuck, Peter Bayer, David Bier, Ingrid R. Vetter and Thomas Schrader

Beilstein J. Org. Chem. **2026,** *22,* 557–567. doi:10.3762/bjoc.22.41

Experimental part

License and Terms: This is a supporting information file under the terms of the Creative Commons Attribution License (https://creativecommons.org/licenses/by/4.0). Please note that the reuse, redistribution and reproduction in particular requires that the author(s) and source are credited and that individual graphics may be subject to special legal provisions.
The license is subject to the *Beilstein Journal of Organic Chemistry* terms and conditions: (https://www.beilstein-journals.org/bjoc/terms)

Table of contents

General information S1

General synthetic procedure S2

Characterization of tweezer conjugates **1**, **2a–f** S2

Molecular modeling S13

Fluorescence polarization titrations S19

Protein expression S19

X-ray crystallography S19

References.. S21

General information

FP measurements: CLARIOstar Plus plate reader (BMG Labtech).

Crystallization: Mosquito crystallization robot (TTP labtech), Formulatrix Rock Imager 1000 (Formulatrix, USA), Eiger2 16M X-ray pixel detector (Dectris, Switzerland).

$^{1}H/^{31}P/^{13}C$ NMR spectroscopy: DMX300 (300 MHz), Avance NEO 400 (400 MHz), DRX500 (500 MHz) und DRX600 (600 MHz). MestReNova-Software Version 9.0.1 from Mestrelab Research.

HRMS: Bruker maXis 4G spectrometer with electrospray ionization (positive/negative mode). Quantitative evaluation with Compass DataAnalysis (Version 4.1) from Bruker.

HPLC: JASCO HPLC-System with Phenomenex column (Luna© 5 µm C18 (2), 100 Å, 250 × 4 mm) and JASCO UV–vis-detector (UV-975) for analytical purposes and monitoring of click reactions.

Chemicals: peptides for click coupling were synthesized by SPPS at GenScript. All other chemicals were purchased from Acros Organics, Alfa Aesar, Sigma Aldrich, or TCI Chemicals and used without further purification. THF was freshly distilled over Na, reactions were routinely conducted under argon atmosphere.

1 General procedure for the synthesis of hybrid tweezers 1, 2a–f

The butynyl tweezer (9–12 µmol, 1.2–1.3 equiv) and the appropriate azido-peptide (7–10 µmol, 1.0 equiv) were dissolved under argon in 3 mL THF/H_2O 1:1 and DIPEA (12 µL, 8.6 mg, 66.8 µmol) was added. Copper(II) sulfate (8.3 mg, 66.8 µmol) and sodium ascorbate (13.0 mg, 65.5 µmol) were each dissolved in 1 mL water, afterwards the two solutions were combined under vigorous stirring. The yellow, cloudy solution was directly added to the tweezer/peptide solution and stirred at room temperature. Reaction progress was monitored by analytical HPLC. After complete conversion the solvents were removed under reduced pressure and the residue was slurried with water and lyophilized. Purification of the crude product was accomplished by preparative HPLC. After subsequent lyophilization the coupling products were obtained as colorless solids.

Deprotonation to the corresponding sodium salts was carried out by dissolving the tweezer in a mixture of water/acetonitrile 1:1 and adding 1 M NaOH (5 equiv). The mixture was stirred for 1 hour at room temperature and afterwards lyophilized. The yield was quantitative.

1.0 Peptide tweezer 1

Similar to all peptide tweezers in this series, already the first protoype **1** did not produce well-resolved NMR signals, most likely due to insertion of a lysine or arginine side chain into the tweezer cavity (self-incusion). We show here the ^{1}H and ^{31}P NMR spectra of the sodium salt of **1** in D_2O.

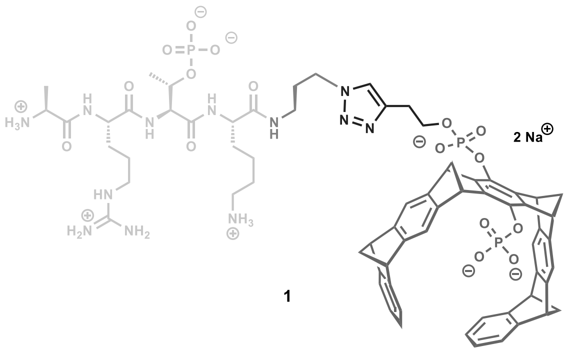

1

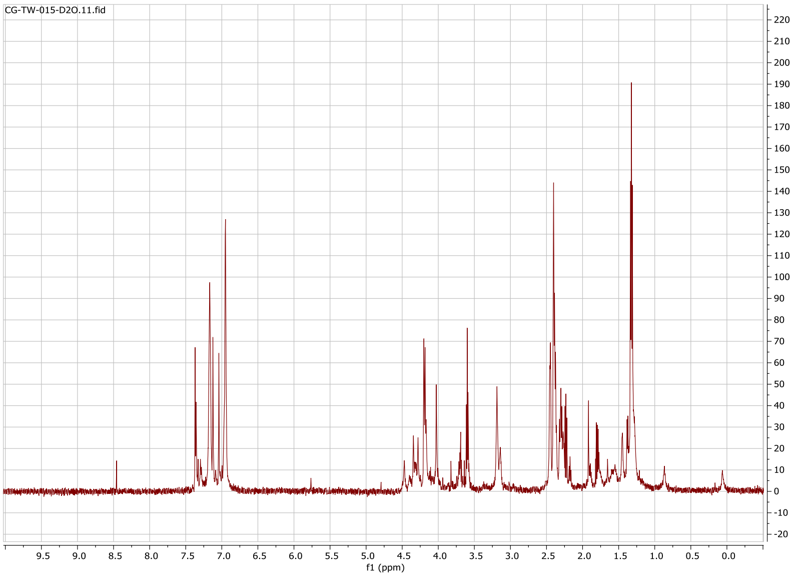

Figure S1: ^{1}H NMR spectrum of **1** (Na^{+} salt in D_2O).

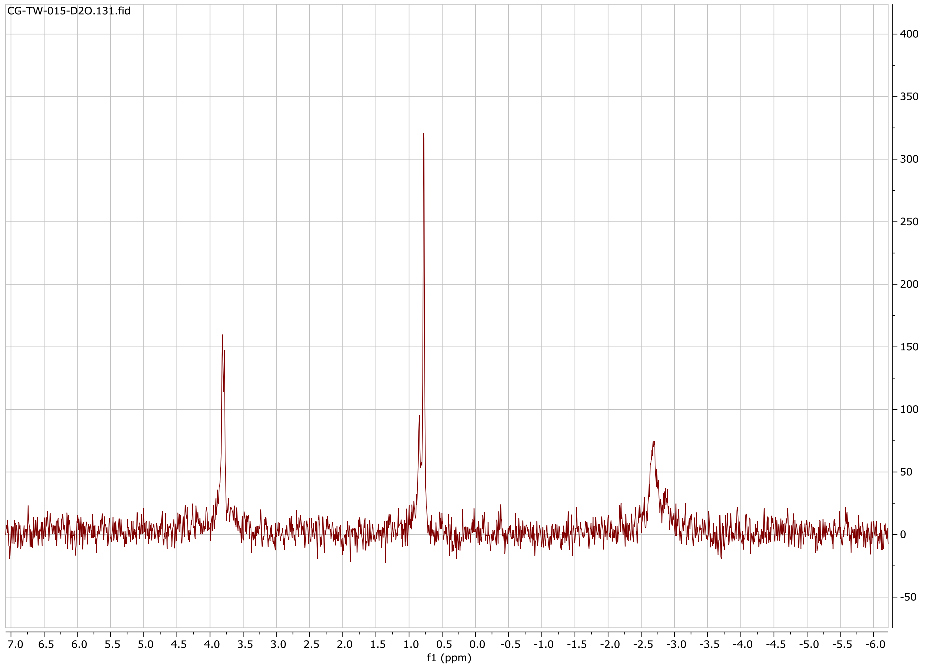

Figure S2: ^{31}P NMR spectrum of **1** (Na^+ salt in D_2O).

1.1 Hybrid tweezer 2a

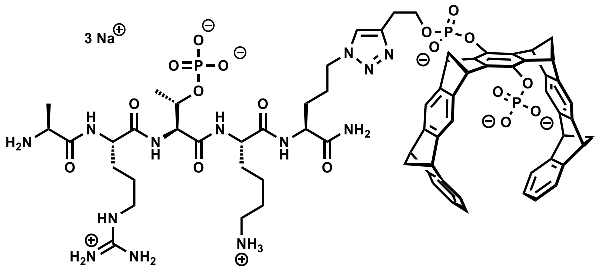

Tweezer–peptide conjugate **2a** was synthesized according to the general procedure using butynyl-tweezer **4a** (8.6 mg, 11.0 µmol, 1.3 equiv) and peptide **3a** (5.9 mg; 8.5 µmol; 1.0 equiv) to obtain the phosphoric acid product with a yield of 43% (5.4 mg; 3.66 µmol).

HRMS; (m/z): $[M+2H]^{2+}$ for $C_{70}H_{84}N_{13}O_{17}P_3H_2$ = 736.7733 (calc.), 736.7754 (obs.); $[M+3H]^{3+}$ for $C_{70}H_{84}N_{13}O_{17}P_3H_3$ = 491.5180 (calc.), 491.5198 (obs.)

^{1}H NMR (600 MHz, DMSO-d$_6$): Even in deuterated DMSO the NMR signals of the tweezer peptide conjugates with lysine and arginine in their sidechains remained very broad, most likely due to self-inclusion of the amino acid sidechains in the tweezer cavities.

^{13}C NMR (151 MHz, DMSO-d$_6$), *δ* [ppm]: 150.18, 147.67, 146.79, 140.97, 137.29, 129.65, 124.52, 121.46, 116.17, 69.78, 68.41, 67.55, 63.86, 59.90, 50.25, 47.87, 35.11, 33.66, 31.28, 30.32, 29.01, 28.73, 28.69, 28.55, 27.87, 27.31, 26.60, 26.56, 25.10, 24.48, 22.09, 13.96, 13.43, 1.15.

^{31}P NMR (162 MHz, DMSO-d$_6$), *δ* [ppm]: -1.53, -3.77.

1.2 Hybrid tweezer 2b

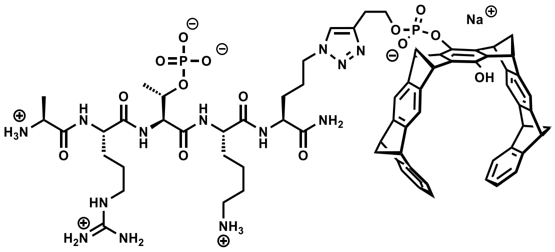

Tweezer–peptide conjugate **2b** was synthesized according to the general procedure using butynyl-tweezer monophosphate **4b** (7.6 mg, 10.4 µmol, 1.2 equiv) and peptide **3a** (6.0 mg; 8.5 µmol; 1.0 equiv) to afford the product with a yield of 47% (5.5 mg; 3.95 µmol).

HRMS; (m/z): $[M+2H]^{2+}$ for $C_{70}H_{83}N_{13}O_{14}P_2H_2$ = 696.7902 (calc.), 696.7850 (obs.); $[M+3H]^{3+}$ for $C_{70}H_{83}N_{13}O_{14}P_2H_3$ = 464.8625 (calc.), 464.8599 (obs.)

1.3 Hybrid tweezer 2c

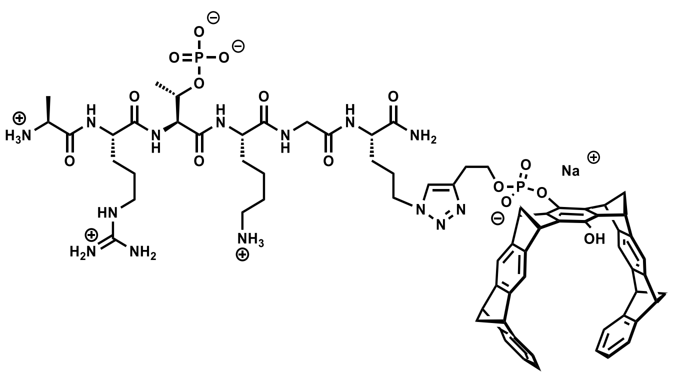

Tweezer–peptide conjugate **2c** was synthesized according to the general procedure using butynyl-tweezer monophosphate **4b** (8.9 mg, 12.8 µmol, 1.2 equiv) and peptide **3b** (8.0 mg; 10.7 µmol; 1.0 equiv) to afford the product with a yield of 33 % (5.1 mg; 3.53 µmol).

HRMS; (m/z): $[M+2H]^{2+}$ for $C_{72}H_{86}N_{14}O_{15}P_2H_2$ = 725.3009 (calc.), 725.3069 (obs.); $[M+3H]^{3+}$ for $C_{72}H_{86}N_{14}O_{15}P_2H_3$ = 483.8679 (calc.), 483.8731 (obs.)

1.4 Hybrid tweezer 2e

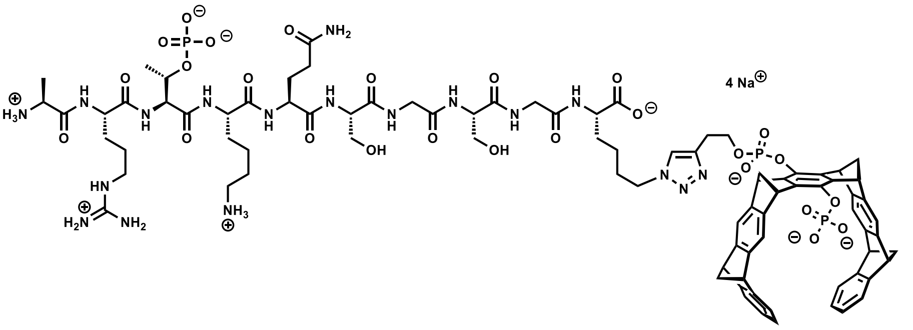

Tweezer–peptide conjugate **2e** was synthesized according to the general procedure using butynyl-tweezer **4a** (6.9 mg, 8.9 µmol, 1.25 equiv) and peptide **3c** (8.0 mg; 7.1 µmol; 1.0 equiv) to obtain the product with a yield of 25 % (3.4 mg; 1.79 µmol).

HRMS; (m/z): $[M+2H]^{2+}$ for $C_{86}H_{109}N_{18}O_{26}P_3H_2$ = 952.8574 (calc.), 952.8592 (obs.); $[M+3H]^{3+}$ for $C_{86}H_{109}N_{18}O_{26}P_3H_3$ = 635.5740 (calc.), 635.5749 (obs.)

1.5 Hybrid tweezer 2f

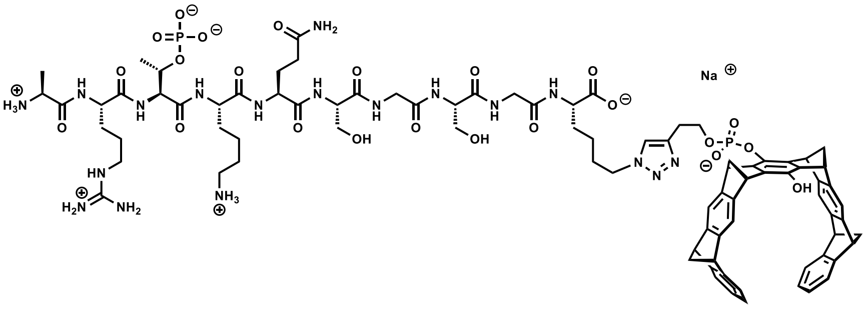

Tweezer–peptide conjugate **2f** was synthesized according to the general procedure using butynyl-tweezer monophosphate **4b** (6.2 mg, 8.9 µmol, 1.25 equiv) and peptide **3c** (8.0 mg; 7.1 µmol; 1.0 equiv) to obtain the product with a yield of 45 % (5.8 mg; 3.18 µmol).

HRMS; (m/z): $[M+2H]^{2+}$ for $C_{86}H_{108}N_{18}O_{23}P_2H_2$ = 912.8743 (calc.), 912.8799 (obs.); $[M+3H]^{3+}$ $C_{86}H_{108}N_{18}O_{23}P_2H_3$ = 608.9186 (calc.), 608.9222 (obs.)

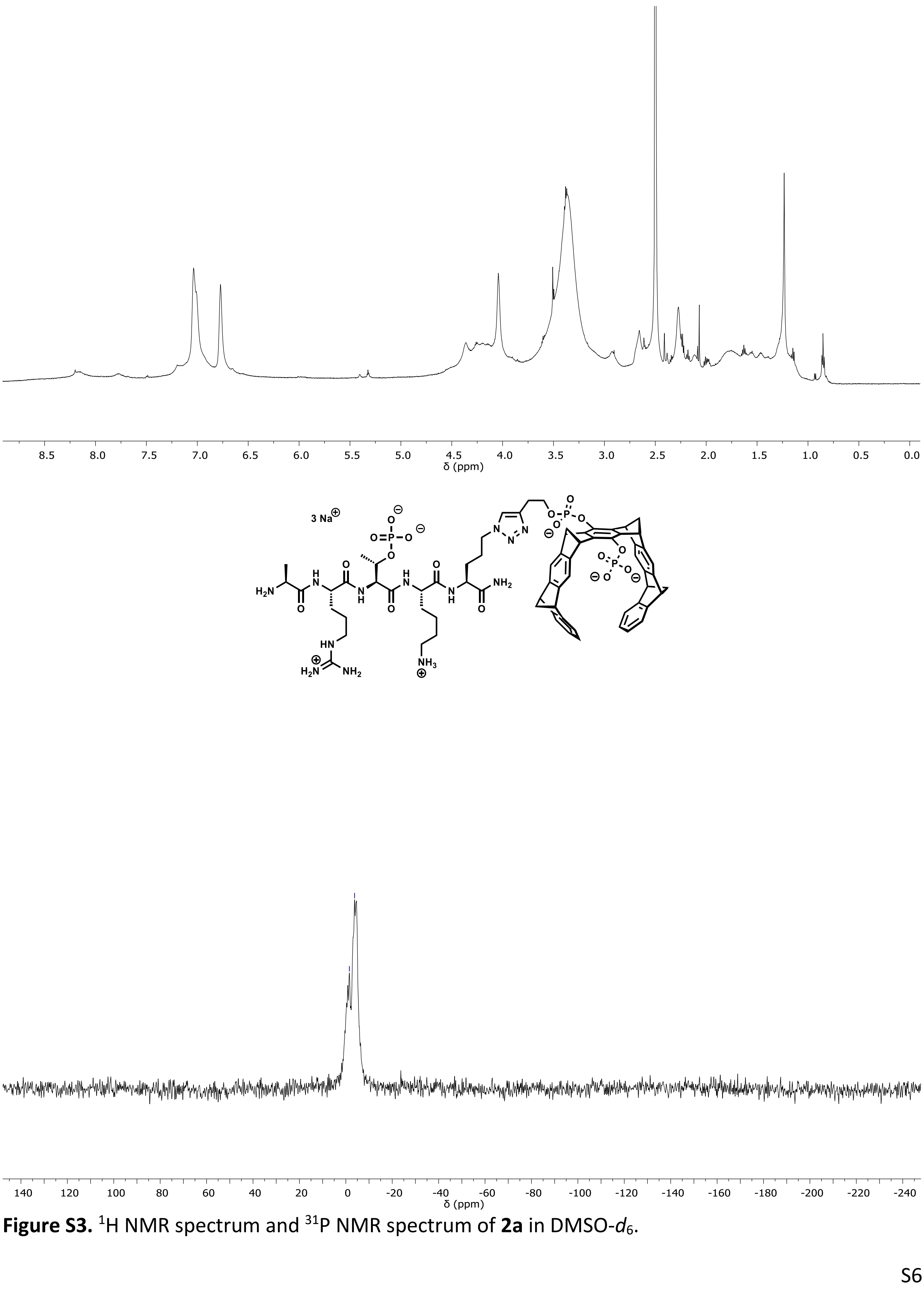

Figure S3. ^{1}H NMR spectrum and ^{31}P NMR spectrum of **2a** in DMSO-d_6.

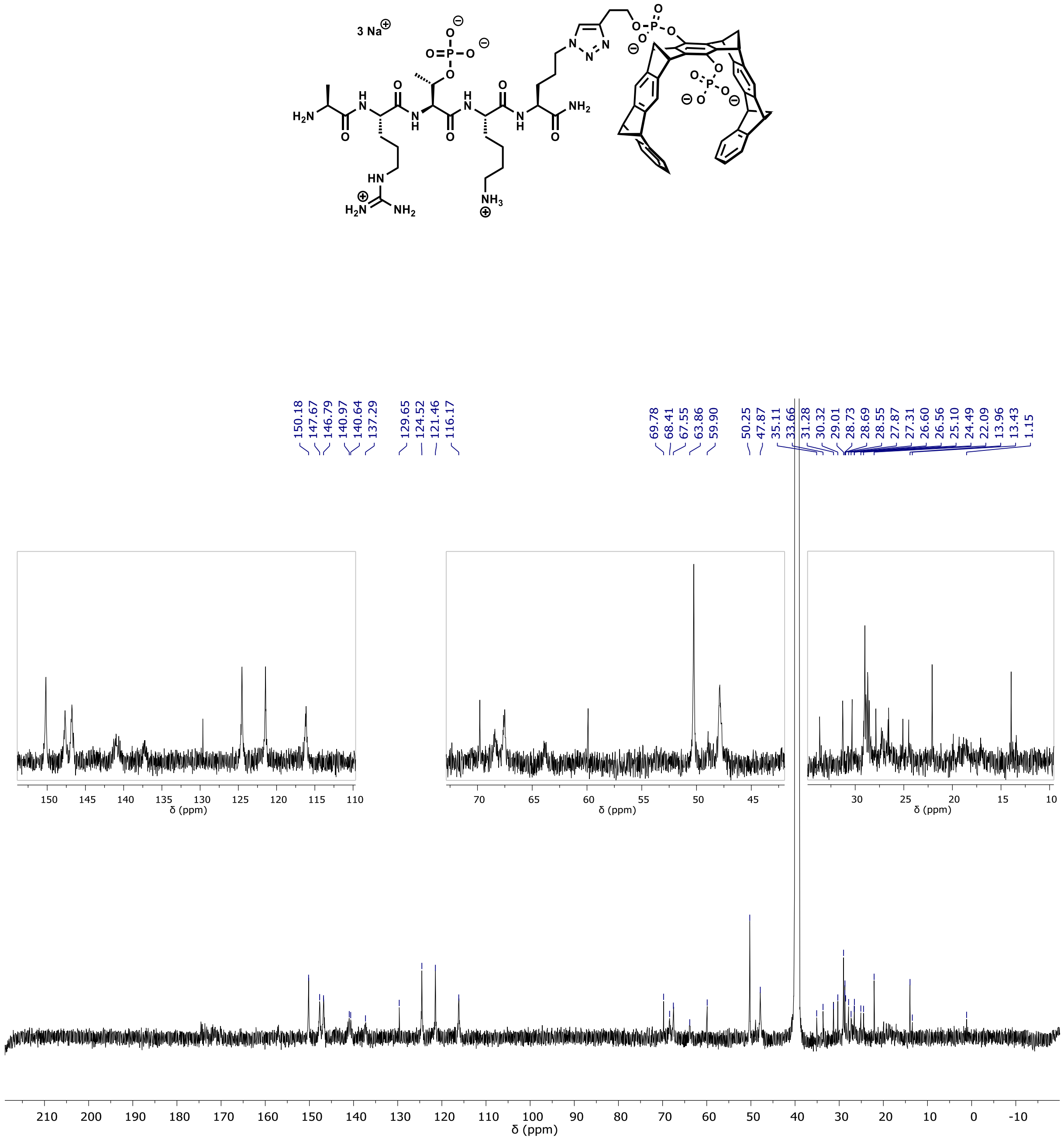

Figure S4. ^{13}C NMR spectrum of **2a** in DMSO-d_6.

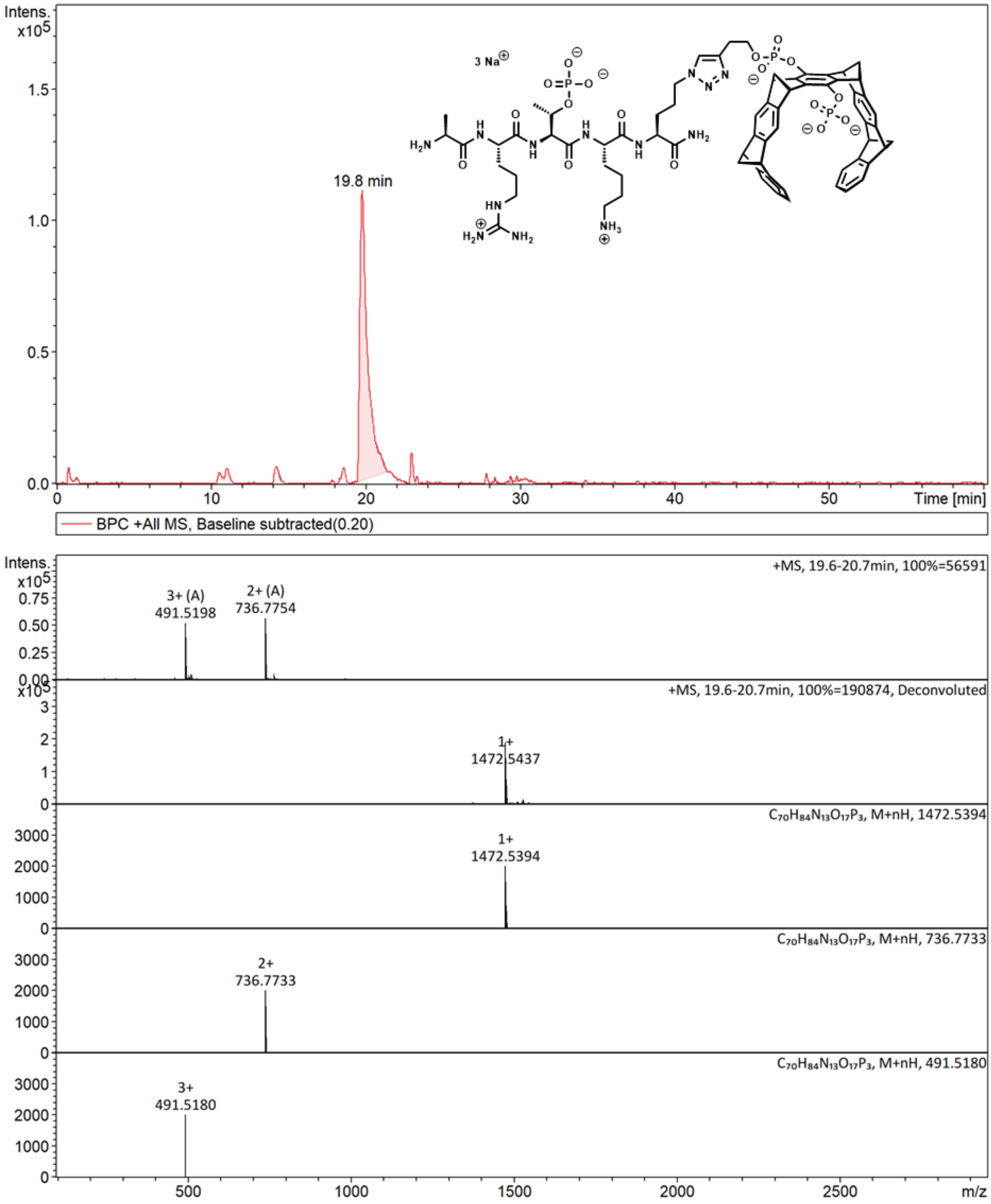

Figure S5. HPLC–MS spectrum of **2a.** Above: chromatogram (TIC). Below: experimental and theoretically expected mass signals for the different ionization levels.

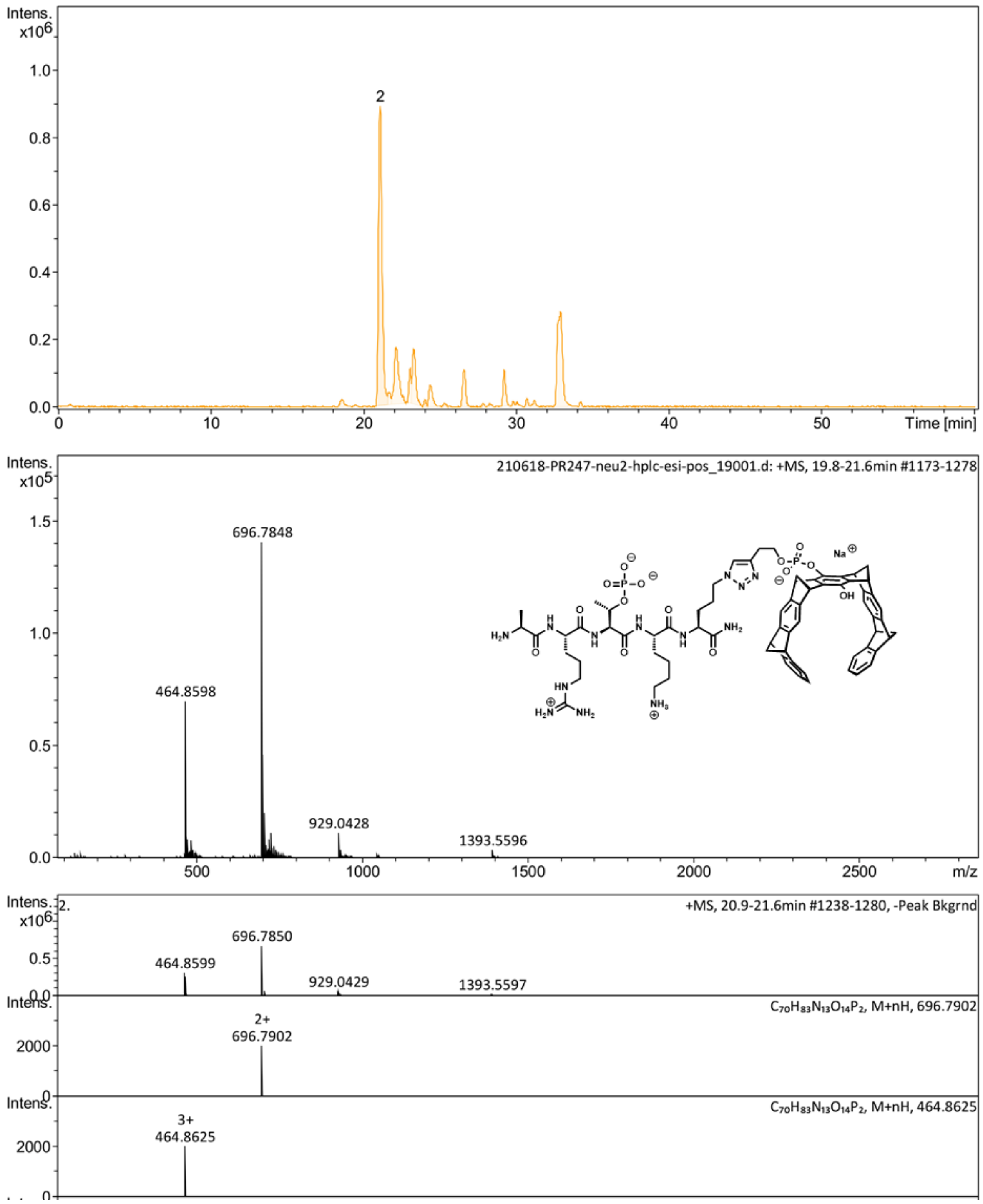

Figure S6. HPLC–MS spectrum of **2b.** Above: chromatogram (TIC). Below: experimental and theoretically expected mass signals for the different ionization levels.

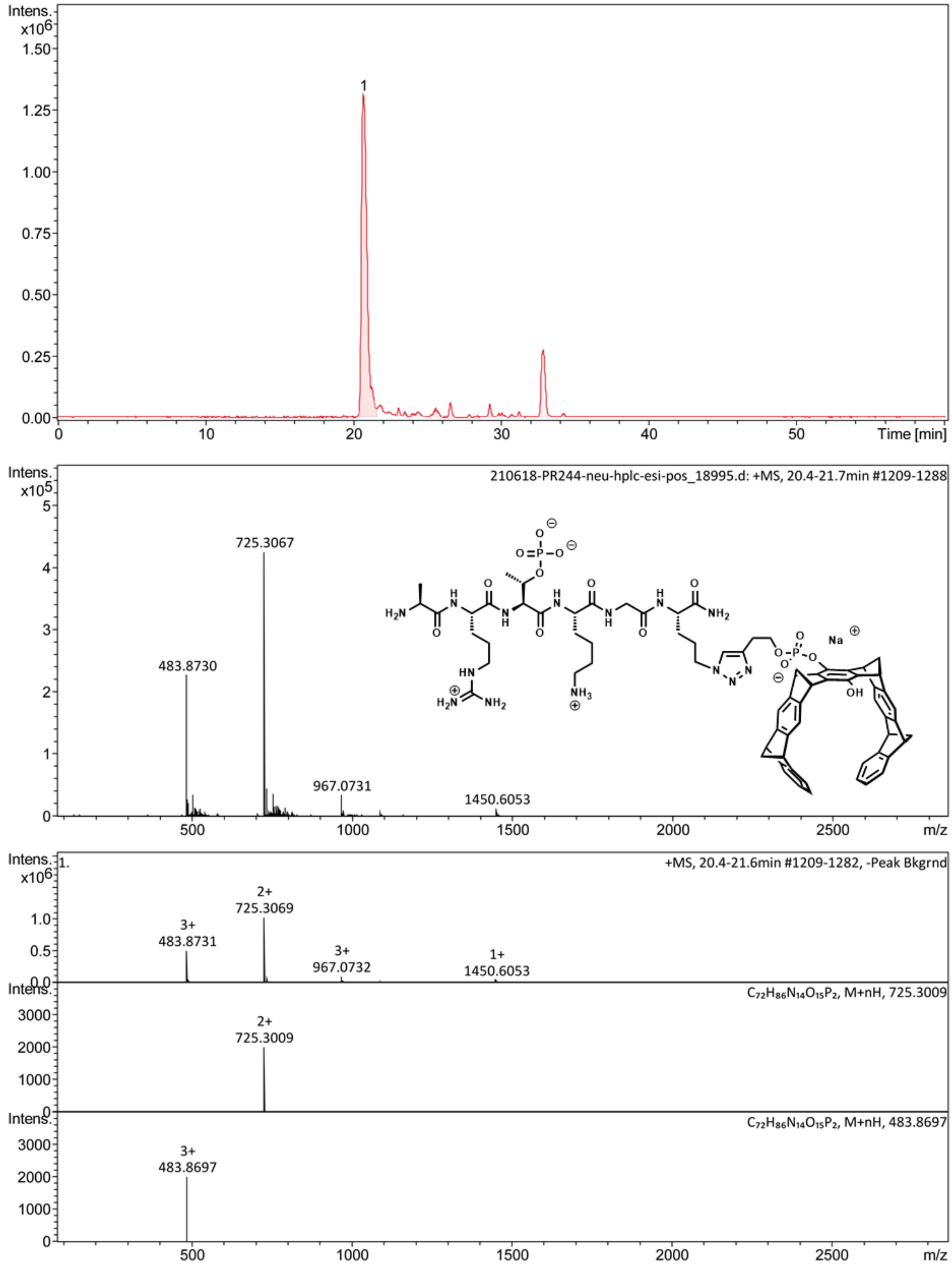

Figure S7. HPLC–MS spectrum of **2c.** Above: chromatogram (TIC). Below: experimental and theoretically expected mass signals for the different ionization levels.

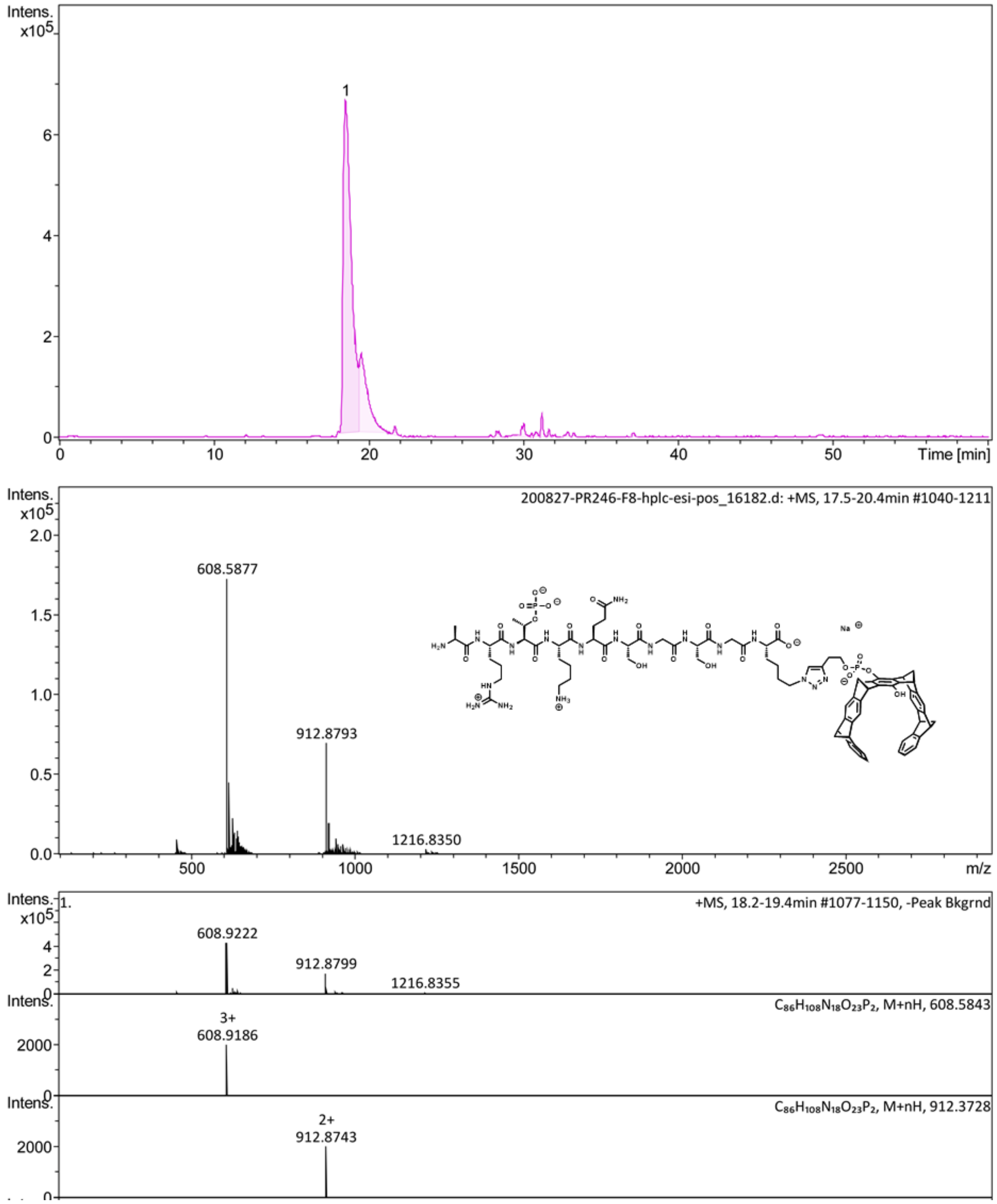

Figure S8. HPLC–MS spectrum of **2e.** Above: chromatogram (TIC). Below: experimental and theoretically expected mass signals for the different ionization levels.

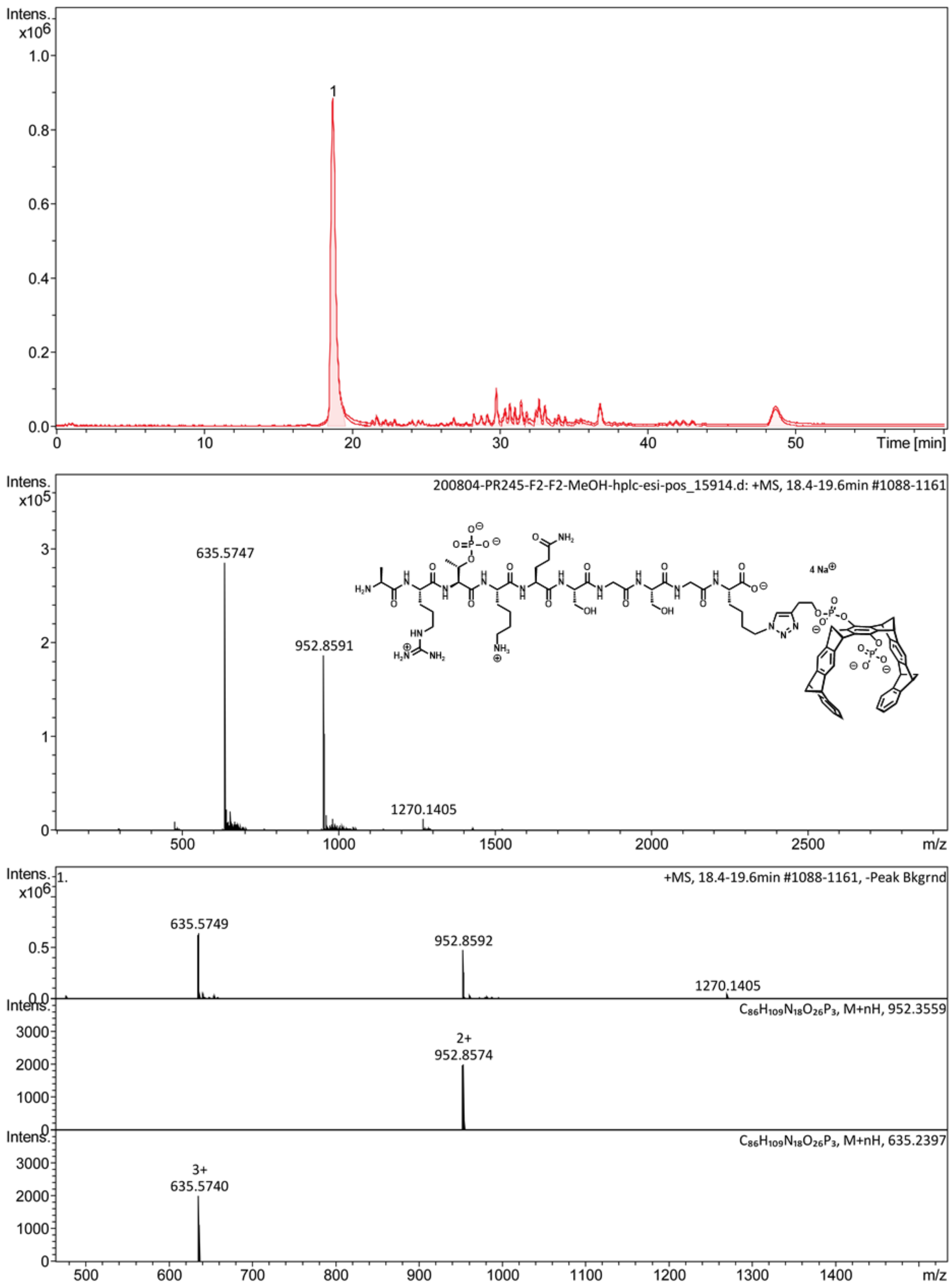

Figure S9. HPLC–MS spectrum of **2f.** Above: chromatogram (TIC). Below: experimental and theoretically expected mass signals for the different ionization levels.

Molecular modeling

Protein pretreatment. In a manner similar to [6], MD simulations were performed with the software package "Desmond" (freeware) and the Maestro suite from Schrödinger, Inc. (release 2019-1). Survivin protein structures were taken from the protein data bank and pretreated using the Protein Preparation Wizard in Schrödinger's suite. Initial protein preparation involved the following steps: bond orders were assigned with reference to the CCD database, hydrogens were added, and missing side chain atoms were completed. All crystallographic water molecules were removed, and heteroatom states were generated at pH 7.3. Protonation states were refined using Epik for pK_a prediction at pH 7.4, and hydrogen bond assignments were optimized using PROPKA at the same pH. A restrained energy minimization was subsequently performed to relax the structure, converging heavy atoms to an RMSD of less than 0.3 Å.

Minimizations. Minimizations of peptides and proteins were performed with the OPLS_2005 force-field, Monte-Carlo simulations were run with 1000 steps. The tweezer–peptide conjugates were minimized separately and then docked onto the protein structure with the histone part occupying its natural binding site.

MD simulations. In a manner similar to [6], all-atom molecular dynamics (MD) simulations were conducted using Desmond (D. E. Shaw Research). The system was solvated in an orthorhombic SPC water box with a 10 Å buffer surrounding the protein complex. The system was neutralized with sodium ions and brought to physiological ionic strength with 150 mM NaCl. Simulations were run under NPT ensemble conditions at 300 K and 1 atm (1013 mbar) for 100 ns, with trajectory frames saved every 1.2 ps, yielding 1000 frames for analysis. All simulations were executed using a NVIDIA GTX 1070 GPU.

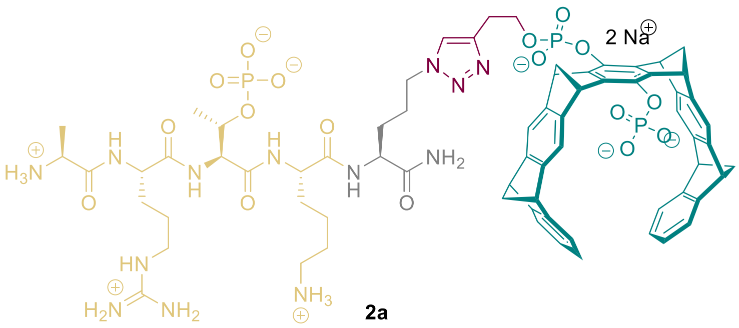

2a

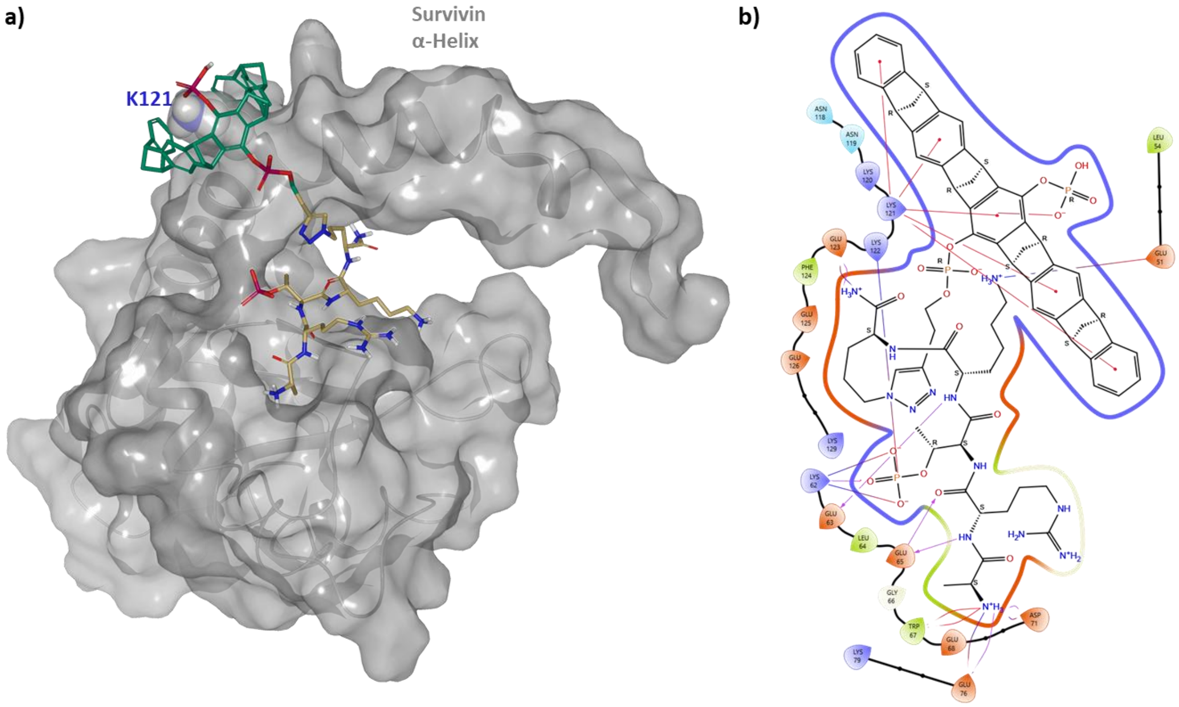

Figure S10. a) MD simulation (final snapshot) of the complex between **2a** (green/yellow) and survivin (PDB: 4A0N) (Desmond, 100 ns, 300 K, 0.15 mM NaCl) with explicit water treatment; K-121 shown as CPK representation. b) Interaction diagram between **2a** and survivin according to the above simulation (100 ns). Arrows symbolize interactions between protein surface and ligand (red – negative charge, blue – positive charge, green – hydrophobic area; light blue – polar area; pink – H bond, red – π-cation).

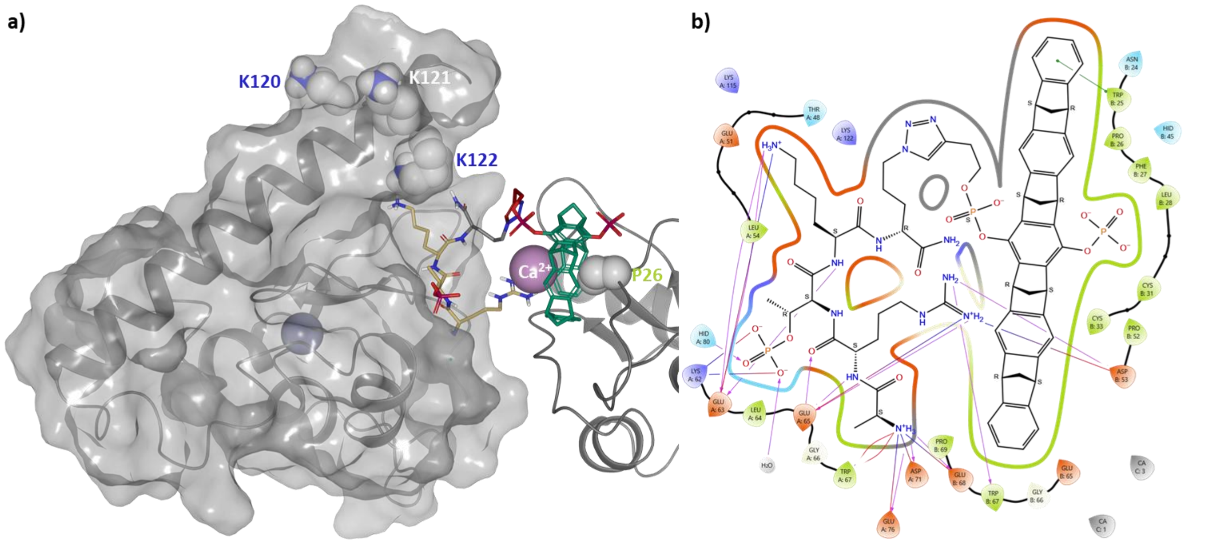

Figure S11. a) ZOOM into one survivin monomer from the crystal structure illustrating the detailed interaction between the protein and tweezer conjugate **2a** (green-red-yellow). The tweezer cavity is occupied in part by a Ca^{2+} ion (pink) and in part by Pro-26 from the other protein monomer (tube representation). b) Interaction diagram of tweezer conjugate **2a** and both protein units in the crystal. Arrows indicate attractive noncovalent interactions between protein and ligand (*protein residues*: red – anionic, blue – cationic, green – hydrophobic, light blue – polar; *noncovalent interactions*: pink – H bond, red – π–cation, green – π–π).

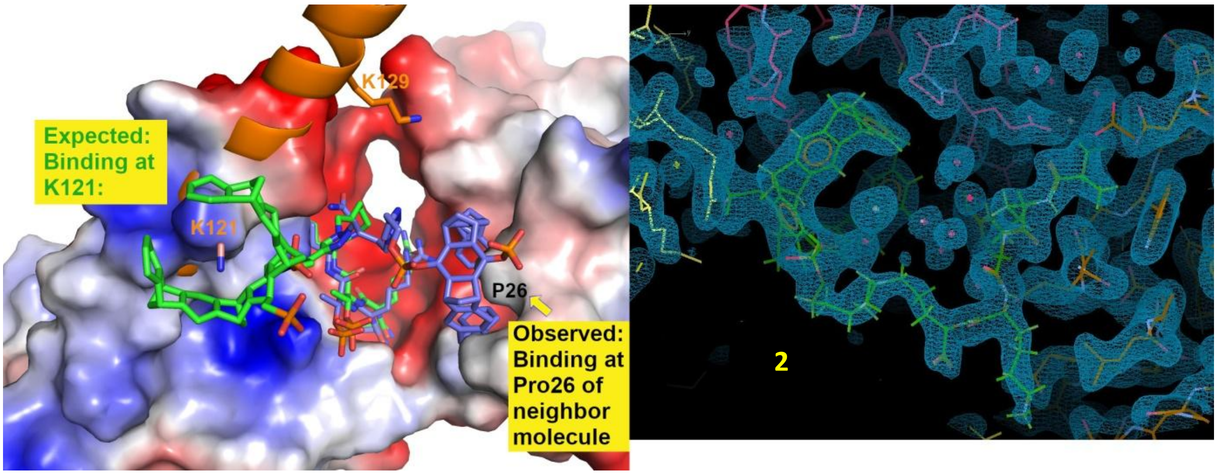

Figure S12. Crystal structure of survivin 1-122/1-127 with the bound hybrid tweezer **2a.** Left: comparison of a model of the expected binding to K121 (green) with the observed binding position (blue). Right: Electron density of the tweezer and the attached H3-peptide.

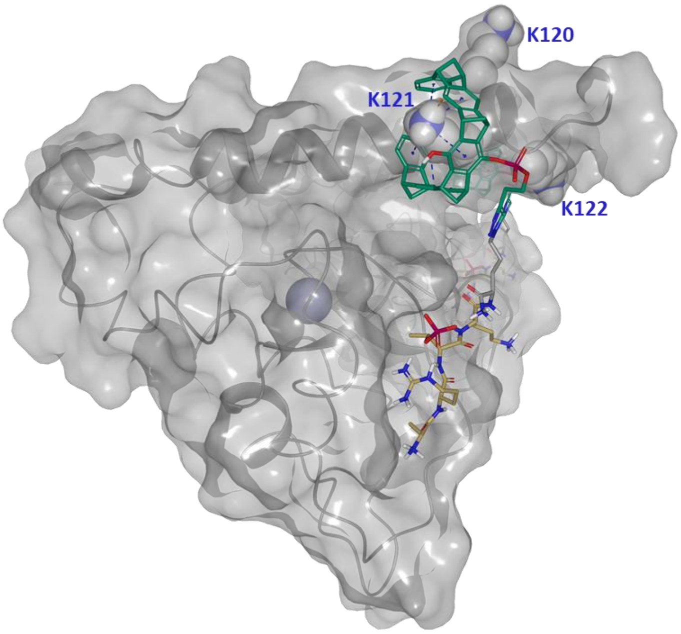

Figure S13. Calculated final structure of the complex formed between tweezer–peptide conjugate **2b** (monophosphate tweezer, green and yellow) on the surface of survivin$_{1–126}$ with encapsulated Lys-121 at the end of the MD simulation (Desmond, 100 ns, 300 K, 0.15 mM NaCl, explicit water solvent). Lys-120, Lys-121 and Lys- 122 are depicted as CPK models. Starting point for this simulation was the crystal structure (Figure S2 and vide infra) of the related tweezer peptide conjugate **2a** (diphosphate tweezer) on survivin.

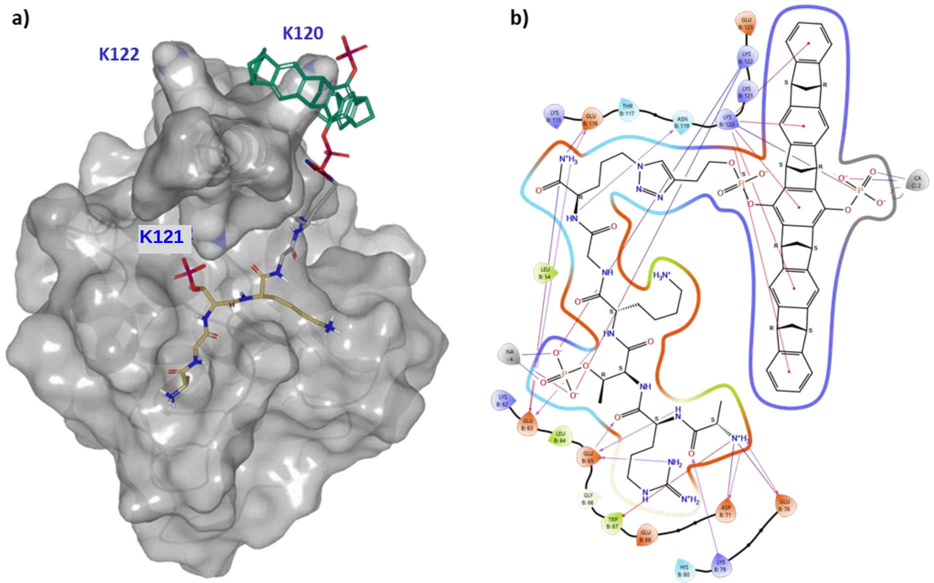

Figure S14. a) Calculated complex structure of tweezer–diphosphate conjugate **2d** (green and yellow) on the surface of survivin 1–127 encapsulating Lys-120 (Desmond, 100 ns, 300 K, 0.15 mM NaCl) with explicit water solvent. (K-121 must be corrected from the middle K-122). The MD simulation started from the crystal structure between tweezer–conjugate **2a** and survivin. b) Interaction diagram for the complex from a). Arrows symbolize attractive interactions between the respective protein parts and ligand areas (protein: red – negative charge, blue – positive charge, green – hydrophobic area, polar – light blue; interactions: pink – H-bonds, red– π–cation interaction).

Fluorescence polarization titrations

In neutral buffer (20 mM HEPES pH 7.5, 300 mM NaCl, 1 mM DTE) wild-type survivin and the overexpressed mutants suvivin$_{1-122}$ and survivin$_{1-127}$ were titrated with aliquots of the FITC-labeled natural N-terminal histone H3 tetrapeptide sequence. The resulting binding curve was analyzed with nonlinear regression and furnished affinity values close to a K_d of 1 µM.

In a competition experiment, the preformed 1:1 complex between survivin and the FITC-labeled histone tetrapeptide was treated with aliquots of the tweezer–peptide conjugate **1**. The resulting IC_{50} value of 29 µM (apparent dissociation constant) corresponds to a K_i value of 122 nM for direct binding between survivin and the tweezer–peptide conjugate.

By contrast, the same experiment conducted with the K-121-A survivin mutant, produced a K_i value for the direct tweezer–survivin interaction of only 22 µM, indicating two orders of magnitude weaker affinity.

Protein expression

Survivin 1-122 and 1-127 were created by introducing a stop codon at the position Ala128 via Gibson cloning in the plasmid pGEX6p2RB with a TEV site so that the resulting construct starts with residue Gly2 and ends with Thr127 (GAPTLPP…FEET-127). The constructs were expressed in *E. coli* BL21 (DE) at 20 °C and spun down at 4500 rpm for 20 minutes. The cell pellets were resuspended in 5 mL lysis buffer (20 mM HEPES pH 7.5, 300 mM NaCl, 1 mM DTE, 1 mM PMSF) and stored at −20 °C. The frozen cell pellet was thawed and 200 µL DNase added, following disruption by a microfluidizer. Afterward, the suspension was spun at 70 000*g* for 30 minutes at 4 °C to gain the protein lysate. The supernatant was filtrated through a filter with pore size of 0.45 µm to get rid of pellet fragments. The lysate as loaded on a 20 ml GSH column and cleaved with TEV protease overnight at 4 °C, following concentration with a 3K filter at 4000 rpm, 4 °C to a final volume of approximately 10 mL. The protein was loaded with a flow rate of 0.5 mL/min on a 320 mL SEC column equilibrated with buffer containing 20 mM HEPES pH 7.5, 300 mM NaCl, and 1 mM DTE. Due to the presence of GST impurities, a second affinity chromatography with a 20 mL GSH column was performed and the flowthrough was concentrated to 10 mg/mL. For storage, the protein solutions were aliquoted, flash-cooled in liquid nitrogen, and stored at −80 °C.

The peptide for the hybrid tweezer **1**, Ala-Arg-pThr-Lys-Orn(N3), was ordered from LifeTein (USA) and clicked to the CLR01 tweezer.

Protein–tweezer co-crystallization

For protein co-crystallization with the hybrid tweezer **1**, equimolar amounts of protein and ligand were used at 10 mM each. The crystallization trials were performed using the sitting drop vapor diffusion technique and crystallization screens from Qiagen (Hilden). 100 nL of the protein solution were mixed with 100 nL reservoir solution and incubated at 4 °C. Crystals of both survivin constructs appeared after 2 days in the JCSG Core III screen in condition C1 (0.1 M imidazole pH 8.0, 0.05 M CaAc, 35% v/v 2-ethoxyethanol) and flash-frozen in liquid nitrogen.

The space group of both crystals is *C*2, but with significantly different unit cells (see Table S1), where one axis of the survivin 1-122 crystal is almost 13 Å shorter due to a slightly different packing caused by the shorter C-terminal helix. Both survivin dimers in the two crystal forms still have the same relative

orientation to each other. A strong blob of density – too large to be water – between the center of the crescent-shaped aromatic tweezer and the linker most likely corresponds to a calcium ion from the crystallization buffer that contains 50 mM calcium acetate.
Datasets were collected at 100 K using an Eiger2 16M detector at the X10SA beamline of the SLS synchrotron in Villigen, Switzerland, integrated using XDS and DIALS [1,2], and scaled with XSCALE [1]. The structures were solved via molecular replacement with PHASER (CCP4 suite) [3] using PDB ID 3UIG as template, both crystal forms have two surviving complexes per asymmetric unit. Refinement with REFMAC (CCP4 suite) [3] and PHENIX [4] and model building with COOT [5] resulted in models with good geometries (Table S1).
The coordinates and structure factors have been deposited with the RCSB with the accession codes 9PTI and pPTH.

Acknowledgements
We thank the beamline staff of X10SA at the Swiss Light Source Paul Scherrer Institute, Villigen, CH, for support, and our colleagues Eckhard Hofmann, Sebastian Oesterlin, and Jan Burnik for help with the data collection.

Table S1. Data collection and refinement statistics.

	**Survivin 1-127** Conjugate **2a (9TPH)**	**Survivin 1-122** Conjugate **2a (9TPI)**
**Wavelength**	0.999891	0.99989
**Resolution range (Å)**	49.97-2.0 (2.071-2.0)	48.85 - 1.8 (1.864 - 1.8)
**Space group**	C 1 2 1	C 1 2 1
**Unit cell (Å/degrees)**	101.5 64.1 75.9<br>90º 141.4º 90º	95.45 63.99 63.08<br>90º 127.61º 90º
**Total reflections**	137147 (13798)	172488 (8498)
**Unique reflections**	20219 (1988)	25433 (1494)
**Multiplicity**	6.8 (6.9)	6.8 (5.7)
**Completeness (%)**	97.98 (96.69)	90.76 (53.79)
**Mean I/sigma(I)**	11.52 (1.11)	10.89 (1.24)
**Wilson B-factor**	32.00	31.88
**R-merge**	0.4275 (1.827)	0.09612 (1.272)
**R-meas**	0.4633 (1.974)	0.1042 (1.401)
**R-pim**	0.1767 (0.7403)	0.03966 (0.5709)
**CC1/2**	0.92 (0.415)	0.997 (0.668)
**CC***	0.979 (0.766)	0.999 (0.895)
**Reflections in refinement**	20204 (1986)	25393 (1490)
**Reflections for R-free**	1010 (99)	1272 (75)
**R-work**	0.2165 (0.3830)	0.2039 (0.4463)
**R-free**	0.2556 (0.3794)	0.2409 (0.4410)
**CC(work)**	0.945 (0.654)	0.953 (0.761)
**CC(free)**	0.938 (0.688)	0.947 (0.708)
**Non-hydrogen atoms:**	2282	2251
**macromolecules**	2000	1924
**ligands**	211	211
**solvent**	71	116
**Protein residues**	242	235
**RMS(bonds)**	0.014	0.014
**RMS(angles)**	1.76	1.76
**Ramachandran:**		
**favored (%)**	97.48	97.40
**allowed (%)**	2.52	2.60
**outliers (%)**	0.00	0.00
**Rotamer outliers (%)**	2.28	1.90
**Clashscore**	4.11	3.51
**Average B-factor:**	45.88	41.01
**macromolecules**	46.80	41.75
**ligands**	39.27	35.25
**solvent**	39.72	39.17

Statistics for the highest-resolution shell are shown in parentheses.

References

[1] Kabsch, W. Automatic processing of rotation diffraction data from crystals of initially unknown symmetry and cell constants, *J. Appl. Crystallogr.* **1993**, *26*, 795.

[2] Winter, G., Waterman, D. G., Parkhurst, J. M., Brewster, A. S., Gildea, R. J., Gerstel, M., Fuentes-Montero, L., Vollmar, M., Michels-Clark, T., Young, I. D., Sauter, N. K. & Evans, G. DIALS: implemantation and evaluation of a new integration package. *Acta Cryst.* **2018**, *D74*, 85-97.

[3] COLLABORATIVE COMPUTATIONAL PROJECT, NUMBER 4. ``The CCP4 Suite: Programs for Protein Crystallography''. *Acta Cryst.* **1994**, *D50*, 760-763.

[4] Adams, P. D., Afonine, P.V., Bunkoczi, G., Chen, V. B., Davis, I. W., Echols, N., Headd,J. J., Hung, L. W., Kapral, G. J., Grosse-Kunstleve, R. W., McCoy, A. J., Moriarty, N. W., Oeffner, R., Read, R. J., Richardson, D. C., Richardson, J. S., Terwilliger, T. C., Zwart, P. H. PHENIX: a comprehensive Python-based system for macromolecular structure solution. *Acta Cryst.* **2010**, *D66*, 213-221.

[5] Emsley, P., Cowtan, Coot, K. Model-building tools for molecular graphics. *Acta Crystallogr.* **2004**, *D60*, 2126-2132.

[6] Figueroa Bietti, A. L., Kauth, A.-M. A., Hommel, K., Blueggel, M., Mohr, L., Niemeyer, F. C., Beuck, C., Bayer, P., Knauer, S. K., Ravoo, B. J., Schrader, T. Photoresponsive Molecular Tweezers Modulate Taspase 1 Activity. *RSC Chem. Biol.* **2025**, *6*, 1837-1847.
